# Power Training Methods Across the Load–Velocity Continuum: Mechanisms, Applications, and Periodization

**DOI:** 10.3390/sports14100464

**Published:** 2026-10-20

**Authors:** Manuel Pinto, João Crisóstomo, Robson Silva, Christopher Kirk, Javier Abián-Vicén, Luís Monteiro

**Affiliations:** 1 CIDEFES, Faculdade de Educação Física e Desporto, Lusófona University, 1749-024 Lisbon, Portugal; jcpcrisostomo@gmail.com (J.C.); luis.monteiro@ulusofona.pt (L.M.); 2 FADEUP, Faculty of Sport, University of Porto, 4200-450 Porto, Portugal; robinho.personal@hotmail.com; 3 Sport and Physical Activity Research Centre, Sheffield Hallam University, Collegiate Crescent, Sheffield S10 2BP, UK; c.kirk@shu.ac.uk; 4 Performance and Sport Rehabilitation Laboratory, Faculty of Sport Sciences, University of Castilla-La Mancha, Avda. Carlos III s/n, 45071 Toledo, Spain; javier.abian@uclm.es; 5 ICPOL Research Center, Higher Institute of Police Sciences and Internal Security, 1300-352 Lisbon, Portugal

**Keywords:** muscular power, resistance training, power training, velocity-based training, strength and conditioning

## Abstract

Muscular power is a critical determinant of athletic performance. Despite the wide range of resistance training methods proposed to enhance power, uncertainty remains regarding their physiological rationale, practical applications, and optimal integration within long-term training programs. This narrative review synthesizes current evidence on the principal resistance training methods for muscular power development and provides an evidence-informed framework for exercise selection and periodized programming. A structured literature search was conducted using PubMed, SPORTDiscus, and Scopus, complemented by additional sources and reference screening. Seventeen studies were retained for the evidence mapping, while systematic reviews, meta-analyses, position stands, and narrative reviews were used to provide conceptual background and contextual interpretation. No formal study-level methodological quality appraisal was performed; therefore, the findings were interpreted as an evidence-informed narrative synthesis rather than an evidence-graded assessment of training-method effectiveness. Current evidence indicates that different resistance training methods target specific regions of the load–velocity continuum through distinct neuromuscular adaptations. Heavy resistance training primarily enhances maximal force production, whereas ballistic training, Olympic weightlifting derivatives, velocity-based training, optimal power load, cluster sets, variable resistance, isometric training, eccentric overload, complex-contrast training, and triphasic training provide complementary stimuli for optimizing muscular power through different approaches. Overall, the evidence supports integrating complementary resistance training methods within a periodized program tailored to the athlete’s training status, sport-specific demands, and competitive calendar to maximize power development.

## 1. Introduction

Muscular power is one of the most important determinants of athletic performance across a wide range of sports [1].

The ability to generate high levels of force within a very short time underpins high-velocity actions such as sprinting, jumping, throwing, striking and rapid changes in direction, making power a key physical quality for success in sports [1,2].

Optimizing muscular power has become a primary objective of modern strength and conditioning programs [3,4,5].

Mechanically, power (measured in watts) is defined as the product of force and velocity (displacement/time). Therefore, improvements in power can be achieved by increasing force production, movement velocity, or the interaction between these variables. However, power production depends not only on the magnitude of force generated but also on the time taken to apply that force. According to the impulse–momentum relationship, impulse reflects the total force applied over time (force × time) and determines changes in momentum, which represents the quantity of motion of a body (mass × velocity). Consequently, changes in momentum result from the impulse produced during movement, highlighting the importance of generating force rapidly [2,6]. Because most sport-specific actions occur within extremely short contraction times—often less than 300 ms and, in sprinting, as little as 80–100 ms—athletes rarely have sufficient time to develop maximal force. Under these conditions, the capacity to produce force quickly, commonly referred to as the rate of force development (RFD), becomes a critical determinant of performance [2]. Although a higher RFD may facilitate greater force production within the limited time available for a sporting action, RFD and impulse represent distinct mechanical variables. RFD describes the rate at which force is developed, whereas impulse reflects the integral of force over time and determines changes in momentum. Thus, a higher RFD does not necessarily result in a greater impulse, as impulse depends on both force magnitude and the duration over which force is applied. These variables are related to performance in actions requiring rapid force production but should not be considered interchangeable with mechanical power [7].

These mechanical constraints explain why different resistance training methods have been developed to target specific components of the load–velocity continuum. Traditional heavy resistance training primarily increases maximal force production, whereas high-impulse exercises, Olympic weightlifting derivatives and plyometric training emphasize movement velocity and rapid force generation. More recently, approaches such as optimal power load (OPL), velocity-based training (VBT), cluster sets, variable resistance training (VRT), and isometric methods have emerged as evidence-based approaches to optimize power development through more individualized and mechanically specific loading [8,9,10].

Numerous studies have investigated the effectiveness of individual training methods, while several systematic reviews and meta-analyses have examined specific modalities or populations; however, the available literature remains fragmented across training approaches [2,11,12]. Consequently, practitioners lack an integrated framework that allows different power-oriented training methods to be interpreted according to their mechanical characteristics, neuromuscular demands, and potential application across the load–velocity continuum. In particular, the literature has not comprehensively integrated the execution velocities associated with different training methods and linked these characteristics to the practical organization of power development within a periodized training process [4,8].

Therefore, the purpose of this narrative review is to synthesize the current evidence regarding the principal resistance training methods used to develop muscular power. The unique contribution of this review is to integrate multiple training methods within a unified load–velocity framework, linking their mechanical and neuromuscular characteristics with practical prescription and application. In addition to examining the physiological mechanisms, practical applications, advantages, and limitations of each method, the review considers how different approaches may complement one another across different phases of power development. Based on this synthesis, an evidence-informed practical framework is proposed to guide the integration of complementary training methods within a periodized program.

## 2. Methods

To analyze and develop this structured narrative review, we first conducted a focused literature search to identify studies addressing the conceptual and mechanistic framework underlying training and methods for muscular power development. This first analysis was conducted using several databases and complementary sources, including Google Scholar, PubMed, SPORTDiscus, and Scopus, as well as forward citation tracking and backward reference screening. This first search also included an analysis of the relationships between force/load and velocity, as well as training periodization and its application to muscular power development. This component considered relevant primary studies, systematic reviews, meta-analyses, position stands, and narrative reviews without restriction by publication date. These sources were used to establish the theoretical background, clarify mechanical and neuromuscular concepts, and provide broader context for the interpretation of training methods, force–velocity relationships, and periodization strategies for power development.

Following this first approach, a second literature search using a structured approach was conducted to descriptively map primary evidence addressing training methods for muscular power development. PubMed, SPORTDiscus, and Scopus were searched because of their broad coverage of biomedical, sports science, exercise physiology, and strength and conditioning literature. The final database search was conducted on 1 August 2026 and was not restricted by publication date. Only articles published in English were considered. The complete search strategies and database-specific search syntax are provided in Supplementary Table S1.

The search strategy combined terms related to muscular power, including “muscular power,” and “power training,” with terms describing training methods and related prescription approaches, including “strength training,” “maximal strength training,” “heavy resistance training,” “isometric training,” “Olympic weightlifting,” “weightlifting derivatives,” “ballistic training,” “plyometric training,” “velocity-based training,” “optimal power load,” “variable resistance training,” “cluster sets,” “eccentric training,” “complex training,” and “triphasic training.” Boolean operators (AND and OR) were used to combine these concepts. Database-specific adaptations of the search syntax were applied where required by the respective database platforms. Training methods or methodological variants identified through the broader narrative literature search or reference tracking, but not represented in the structured database search, were considered only for narrative contextualization and were not included in the structured evidence mapping.

For the evidence mapping, studies were considered relevant when they investigated a training method or related training strategy intended to develop muscular power and reported execution velocity, load (%), or performance-related outcomes relevant to power development. Studies that did not meet these relevance criteria were not retained for the evidence mapping. Primary experimental studies were the principal evidence unit for this mapping, while secondary evidence sources identified through the broader narrative search were retained for conceptual interpretation and contextualization. These criteria were used to define the scope of the evidence mapping and to support a transparent screening and selection process, rather than to constitute a systematic review protocol.

The evidence-mapping process involved screening retrieved records for relevance to the objectives of the evidence mapping, followed by assessment of potentially relevant full-text articles. Two reviewers independently extracted the relevant study characteristics and outcome data using a standardized extraction framework and cross-checked the extracted information against the original publications. Any discrepancies were resolved through discussion and consensus. This structured process was used to support the descriptive evidence mapping and was not intended to constitute a systematic or scoping review of the broader literature. Additional relevant publications were identified through backward reference screening of key articles and were considered according to the same relevance criteria when contributing to the evidence mapping.

After removal of duplicates, titles and abstracts were screened, followed by full-text assessment of eligible articles. A total of 17 studies met the predefined evidence-mapping criteria and were retained for mapping (Table 1).

No formal study-level risk-of-bias or methodological quality appraisal tool was applied. Instead, the evidence was interpreted qualitatively, taking into consideration study design (experimental studies), sample characteristics (healthy adults), training variables (load or velocity), and outcome measures (power-related outcomes). This approach was adopted because the primary purpose of the mapping was to characterize and organize the available evidence across different training methods and their influence on power development, rather than to produce evidence-graded comparative effectiveness estimates. Accordingly, the evidence mapping was used to organize and contextualize the available primary evidence rather than to establish a formal evidence hierarchy or graded synthesis.

These studies were then mapped according to the primary training method investigated, including heavy resistance training, ballistic training, plyometric training, Olympic weightlifting, variable resistance training, isometric training, eccentric overload training, velocity-based training, optimal power load, cluster sets, complex-contrast training, and triphasic training. When a study investigated more than one method, it was mapped to each relevant method.

Evidence from the broader narrative synthesis and the mapping was integrated qualitatively. For each method, findings were considered in relation to physiological and mechanical mechanisms, training variables, practical applications, advantages, limitations, and its potential positioning within the load–velocity framework. Differences in population, exercise selection, outcome measures, and methodological design were considered when interpreting the findings. No quantitative pooling or meta-analysis was performed.

A narrative review design was selected for two interrelated reasons. First, the objective was to identify the current methods used for muscular power development, with particular attention to what is documented regarding load and velocity and how these variables are characterized across different training methods. Several systematic reviews and meta-analyses have already addressed important aspects of muscular power development and load prescription [2,9,12], but these reviews do not specifically synthesize the available evidence regarding execution velocity across the different methods.

Second, the present contribution aims to develop a unifying conceptual model for the application of different methods during the different phases of muscular power development within periodization. The aim is to integrate the available methods identified in the literature into a practical framework for application across different periodization phases. The added value of this review, therefore, lies in cross-domain integration and the development of a practitioner-oriented conceptual framework. The absence of formal quality appraisal is acknowledged as a limitation of the narrative design.

## 3. Fundamental Concepts of Muscular Power

### 3.1. Definition of Muscular Power

Muscular power represents the rate at which mechanical work is performed and can be expressed as the product of force (measured in Newtons) and velocity (measured in meters per second) [30,31]. Within an individual muscle, as contraction velocity increases, the amount of force that can be generated generally decreases, whereas higher forces are typically produced at lower contraction velocities. At an organism level, maximal power is achieved through an optimal interaction between force and velocity rather than by maximizing either variable independently. This concept highlights that athletes may achieve similar power outputs through different combinations of force and movement velocity, depending on their strength level, training background, and the mechanical characteristics of the exercise performed [12].

From a physiological perspective, maximal power production is influenced by multiple neuromuscular factors. Muscle cross-sectional area, fiber-type composition, fascicle length, pennation angle, tendon stiffness, motor unit recruitment, firing frequency, inter- and intramuscular coordination, and the efficiency of the stretch–shortening cycle (SSC) all contribute to an individual’s capacity to generate power. Furthermore, the expression of muscular power is affected by the specific movement pattern, contraction type, external load and the time available to produce force, demonstrating that power is a highly task-specific physical capacity rather than a single generic quality [9].

It is important to distinguish mechanical power from related mechanical and performance variables. Likewise, outcomes such as jump height, sprint performance, change-of-direction performance, maximal strength, and RFD should not be interpreted as direct measures of mechanical power unless power is specifically quantified.

While maximal strength is considered a fundamental prerequisite for power development, increasing strength alone does not necessarily maximize power output. Numerous studies have demonstrated a strong relationship between maximal strength and power production, particularly in weaker or less-trained individuals, where improvements in strength generally translate into substantial gains in power. However, as athletes become stronger, this relationship gradually weakens, and further improvements in power require more specific training stimuli that enhance the ability to express force within the limited time available during sporting movements. In this context, improvements in RFD may contribute to an enhanced capacity to generate force rapidly, particularly during tasks characterized by short contraction times. Consequently, maximal strength should be viewed as the foundation upon which more specialized power-training methods are subsequently developed [8,12,32].

Although RFD, impulse, and power are related mechanical variables, they represent distinct aspects of force production and should not be considered interchangeable. RFD describes the rate at which force is developed, whereas impulse reflects the accumulation of force over time. Power, in contrast, reflects the rate of mechanical work and is determined by the interaction between force and movement velocity. Consequently, an improvement in RFD or impulse do not necessarily translate into enhanced power. Similarly, improvements in a single mechanical variable should not be assumed to translate automatically into enhanced performance across all sporting actions, as the relevance of each variable depends on the mechanical and temporal demands of the specific task.

### 3.2. Load–Velocity Relationship

**Figure 1:**
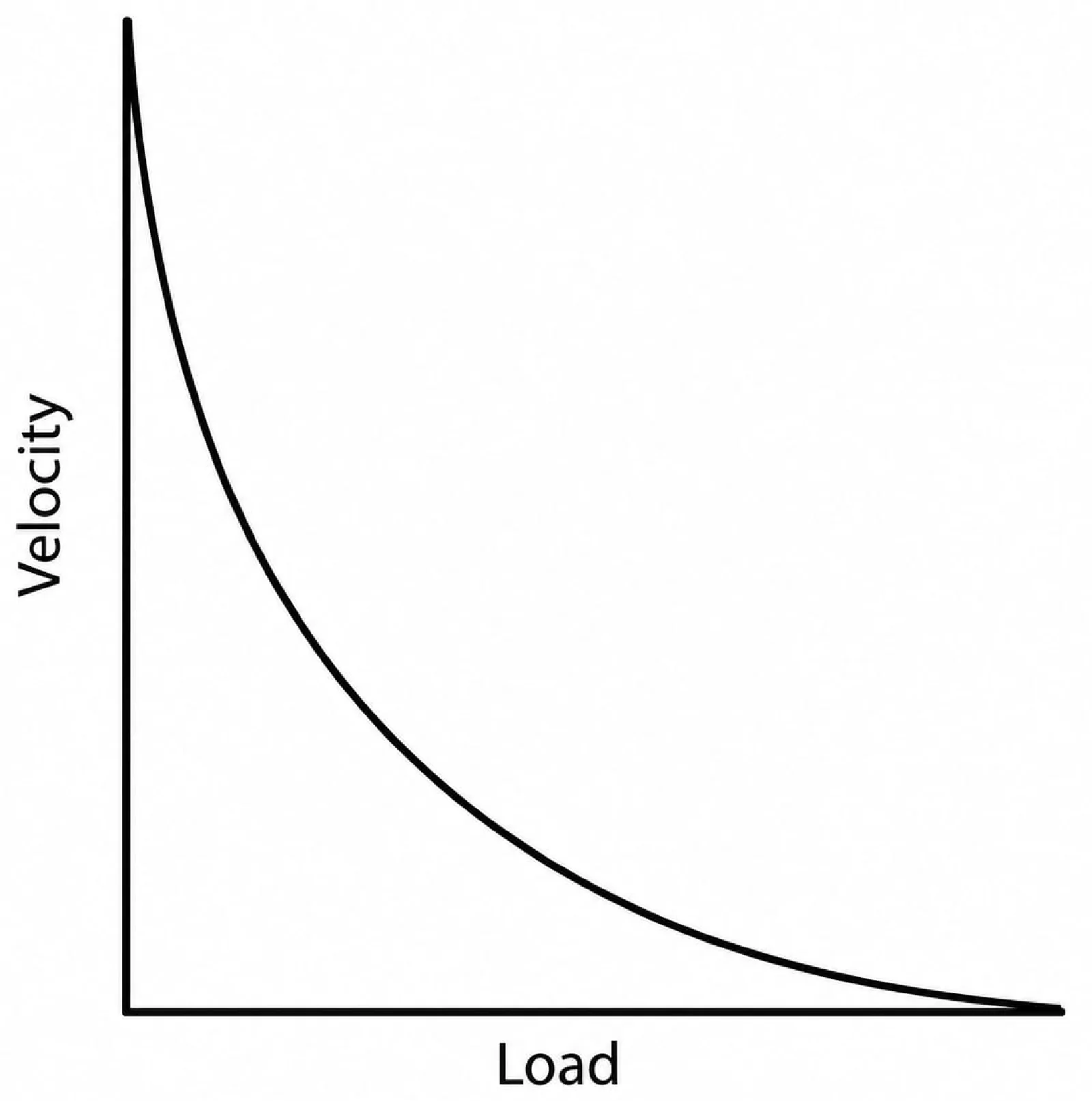
Conceptual illustration of the load–velocity relationship. The curve is schematic and is intended to illustrate the theoretical inverse relationship between load and movement velocity; it does not represent empirically estimated quantitative data.

The force–velocity relationship describes the inverse association between force and contraction velocity, whereby an individual muscle produces greater force at lower shortening velocities, whereas force production progressively decreases as contraction velocity increases (Figure 1). The theoretical basis of this relationship has been extrapolated to whole-body movements such as weightlifting to suggest training prescriptions, i.e., heavier loads result in lower movement velocities, whereas lighter loads allow greater movement velocities, which may contribute to improved power. This concept has been explored in the training literature using the terms ‘force–velocity relationship’ [33] and ‘load–velocity relationship’ [34,35], which are related but distinct constructs and should not be considered strictly interchangeable. The force–velocity relationship refers to the biomechanical relationship between force production and contraction velocity, whereas, in whole-body resistance exercises, practitioners typically manipulate external load and observe the corresponding changes in movement velocity. Therefore, the term load–velocity relationship may provide a more precise description when referring specifically to the relationship between prescribed external load and movement velocity during resistance exercise. Moreover, load–velocity characteristics may vary according to individual and methodological factors, including sex [35]. Since muscular power reflects the interaction between force and velocity, maximal power is generally observed at intermediate combinations of these variables rather than at their respective extremes. However, the specific load and velocity at which maximal power occurs are not universal and depend on factors such as the exercise performed, the contribution of body mass to the movement, the method used to quantify power, and whether mean or peak power is considered [2,8,35].

The simplified load–velocity relationship discussed and illustrated in the present section primarily refers to the concentric phase of dynamic resistance exercise, in which prescribed external load is examined in relation to movement velocity. Eccentric and isometric actions represent mechanically distinct conditions and are therefore not adequately described by this same simplified framework.

**Figure 2:**
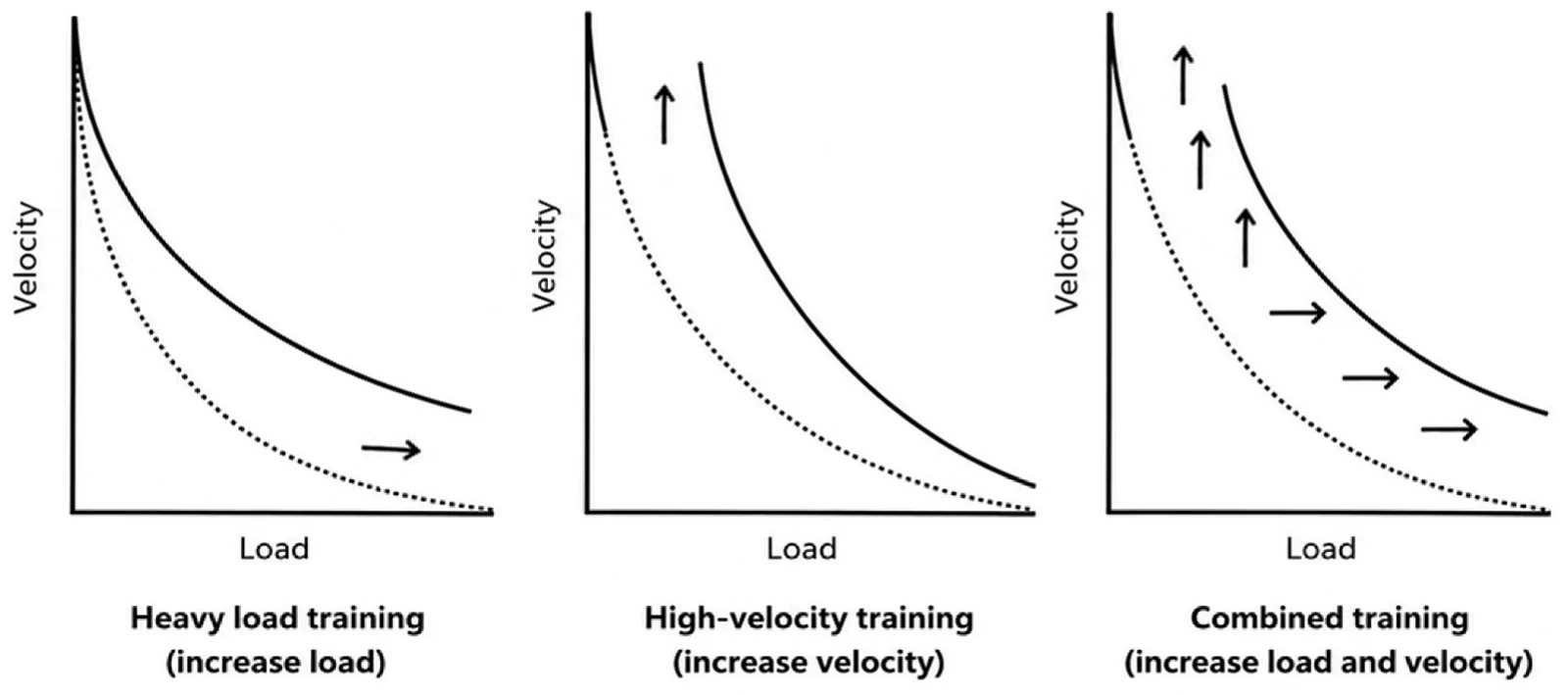
Conceptual illustration of the potential effects of heavy-load, high-velocity, and combined resistance training on the load–velocity relationship. The illustrated shifts are schematic and theoretical and should not be interpreted as empirically estimated effect curves or quantitatively validated adaptations.

The practical implications of the load–velocity relationship are substantial for strength and conditioning practitioners. Athletes who predominantly train with heavy loads improve their ability to generate force under low-velocity conditions, whereas those exposed to lighter, high-velocity training may enhance power production through adaptations that favour force production at higher movement velocities [14]. Consequently, different loading strategies induce specific adaptations along distinct regions of the load–velocity curve, emphasizing that no single training method can optimize power across the entire spectrum of load and velocity demands (Figure 2) [2,9].

**Figure 3:**
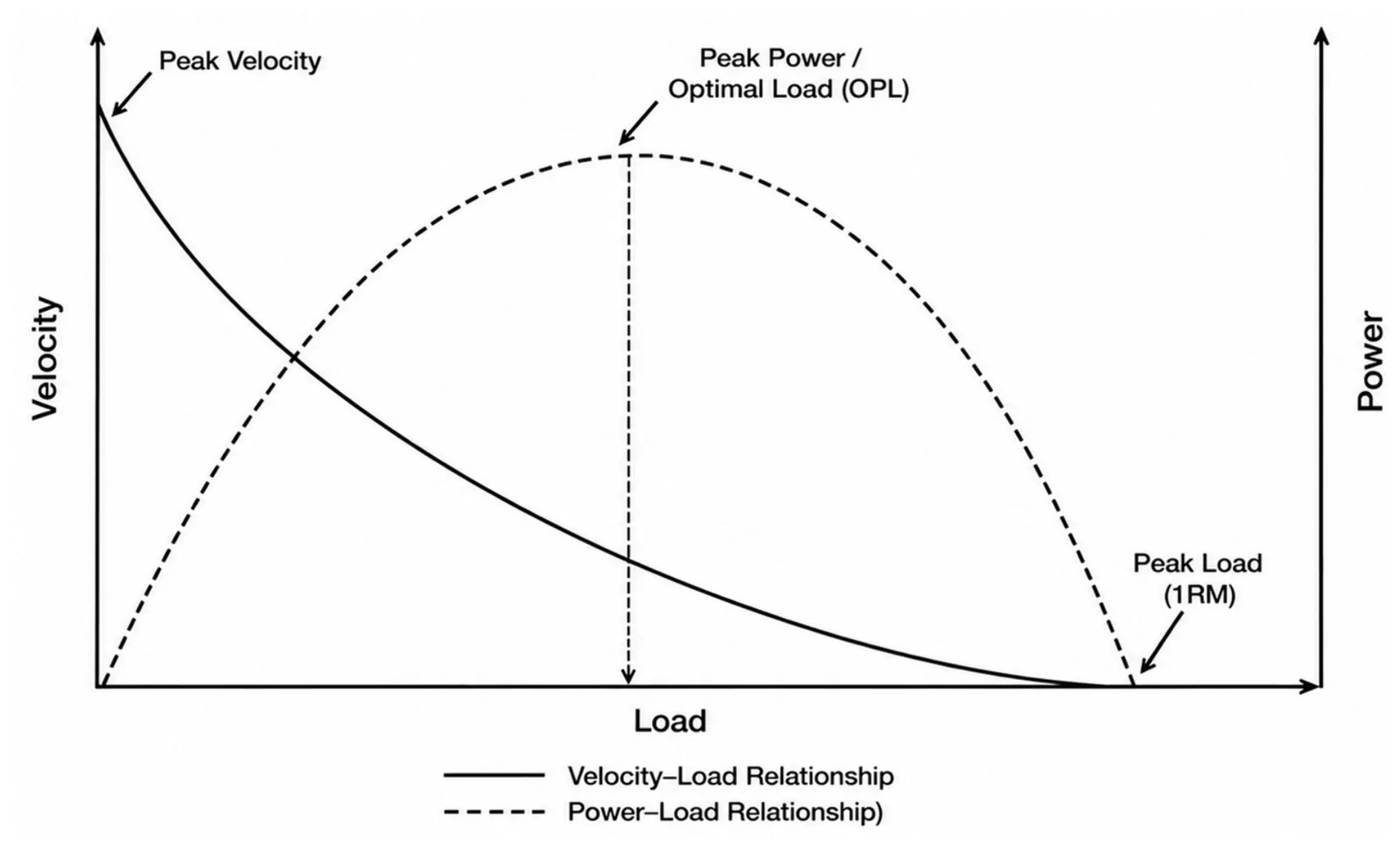
Conceptual illustration of the theoretical load–velocity and power–load relationships, including Optimal Power Load (OPL), where maximal power output is theoretically achieved through an optimal interaction between force and movement velocity. The curves are schematic and do not represent empirically estimated quantitative relationships.

The improvements in maximal strength shift the load–velocity curve upward, enabling athletes to produce greater force across a wide range of movement velocities. This adaptation is particularly important in weaker or less-trained individuals, where increases in maximal strength are strongly associated with improvements in maximal power output. However, as athletes become stronger, further increases in maximal strength contribute progressively less to power development, highlighting the need for more velocity-specific and movement-specific training interventions [2,9]. The task-specific nature of power production has important implications for exercise selection and training transfer. The ability to generate maximal power during dynamic, multi-joint movements depends substantially on the mechanical and coordinative characteristics of the movement performed. Consequently, the exercises selected within a power-training program may influence not only the magnitude of performance improvements but also the specific neuromuscular adaptations that are developed. Training movements that share relevant mechanical characteristics with the target sporting action may therefore provide a greater potential for transfer to high-intensity performance [9,36]. Load selection plays a critical role in power-oriented training because different loading conditions induce distinct neuromuscular adaptations. In general, heavy loads (typically ≥80% 1RM) predominantly improve maximal strength and force production, whereas lighter loads (approximately 0–60% 1RM) are more effective for enhancing movement velocity, RFD, and neuromuscular activation. Between these loading ranges lies the OPL, defined as the load that maximizes power output in a given exercise. However, the OPL is not universal and depends on several methodological and biomechanical factors, including the exercise performed, whether the load is expressed as external barbell load or system load (body mass plus external load), the power metric considered (e.g., peak or mean power), and the athlete’s strength level and movement characteristics. Consequently, maximal power has been reported to occur at approximately 30% of 1RM during the jump squat, around 30–45% of 1RM during the bench throw, and approximately 70–80% of 1RM during Olympic weightlifting derivatives [9]. These theoretical relationships are illustrated in Figure 3.

Overall, the load–velocity relationship provides the scientific framework for understanding why multiple resistance training methods are required to maximize muscular power. Rather than favoring a single loading strategy, effective power-training programs should manipulate load, movement velocity, and exercise selection to target different regions of the load–velocity continuum according to the athlete’s characteristics and the specific performance demands of the sport.

However, the load–velocity continuum should be considered a useful conceptual framework rather than a strict physiological categorization system. Different training methods may influence multiple neuromuscular qualities simultaneously, and athletes’ adaptations are not determined exclusively by external load or movement velocity. Therefore, the positioning of individual training methods along the continuum should be interpreted as a practical representation of their predominant mechanical characteristics rather than as a rigid classification of their physiological effects.

### 3.3. Neuromuscular Determinants of Power

Muscular power results from the coordinated contribution of muscular morphology, neural activation, tendon properties, and movement mechanics. Consequently, improvements in power require training interventions that target these multiple determinants simultaneously [12].

Neural factors play a central role in rapid force production performance. The rapid recruitment of high-threshold motor units, increased motor unit firing frequency, improved synchronization, and enhanced inter- and intramuscular coordination enable force to be produced more quickly. These neural adaptations contribute directly to improvements in the RFD, allowing athletes to reach high force levels within the short time available [2].

Muscle morphology further influences power production. Larger muscle cross-sectional area increases the capacity to generate force, whereas muscle architecture—including fascicle length and pennation angle—affects contraction velocity and force transmission. In addition, fiber-type composition is particularly relevant, as Type II muscle fibers possess greater shortening velocities, higher force-generating capacity, and in turn superior impulse characteristics compared with Type I fibers. This is also influenced by motor neuron type and activity, with Type 1 fibers being innervated by smaller neurons with slower conduction velocities. Type 2 fibers in contrast are innervated by larger motor neurons capable of higher velocity conduction [37]. Adaptations to both neural and muscular elements of a motor unit are therefore achievable and required. These structural characteristics help to explain force and power differences between athletes, while providing the mechanical foundation upon which neuromuscular adaptations to training occur [9].

The mechanical properties of tendons also contribute substantially to rapid force production performance. Increased tendon stiffness improves the efficiency of force transmission from muscle to bone, while simultaneously enhancing the storage and release of elastic energy during SSC activities. The SSC further contributes to rapid force production by coupling eccentric and concentric muscle actions, facilitating the storage and subsequent reutilization of elastic energy and potentially enhancing force and power output compared with concentric-only actions [12,14]. Consequently, tendon adaptations facilitate greater force production and can improve movement efficiency during sporting actions requiring rapid force production transitions between eccentric and concentric muscle contractions [9].

Taken together, these findings demonstrate that optimal power development cannot be achieved by increasing strength or movement velocity in isolation. Instead, successful power-training programs should simultaneously develop maximal force capacity, neural activation, rapid force production, muscle architecture, and movement-specific coordination. Understanding these neuromuscular determinants provides the physiological rationale for the wide range of resistance training methods that will be discussed in the following sections of this review.

## 4. Power Training Methods

Power-oriented training encompasses several complementary approaches that differ in their purpose and application. First, training modalities represent the primary exercise interventions used to develop power, including heavy resistance training, ballistic training, Olympic weightlifting, plyometric training, eccentric overload training, and isometric training. Second, prescription and monitoring strategies, such as OPL and VBT, are used to individualize and optimize training intensity. Third, set configurations, such as cluster sets, modify the organization of repetitions and rest intervals to influence training adaptations. Finally, periodization models, such as triphasic training, provide a framework for organizing these methods over time. The following sections describe each of these approaches according to its role in power development.

It should be acknowledged, however, that the quantity and methodological strength of the available evidence vary substantially across these approaches. While some methods are supported by a relatively extensive body of experimental research, others are supported by a smaller number of studies or more preliminary evidence. Therefore, the organization of the following sections does not imply equivalent levels of evidential support or effectiveness, and the findings for each approach should be interpreted according to the characteristics and extent of the available evidence.

### 4.1. Training Modalities

#### 4.1.1. Heavy Resistance Training

Heavy resistance training is a primary resistance training method used to develop maximal strength and may provide an important foundation for power-oriented training. This method typically involves lifting heavy external loads (≥80% of 1RM) with maximal intent to maximize force production. Because maximal strength represents the upper limit of force production, increasing maximal strength may provide an important foundation for subsequent power-oriented adaptations. However, its contribution depends on the athlete’s initial strength level, the task performed, contraction time, and the performance outcome being assessed [9,14,38].

The adaptations induced by heavy resistance training are mediated through both neural and morphological mechanisms. Heavy-load resistance exercise may enhance the recruitment of high-threshold motor units, increase voluntary activation, and improve intermuscular coordination, leading to greater maximal force production. Chronic exposure to heavy loading also promotes skeletal muscle hypertrophy and favourable adaptations, including increased muscle thickness and pennation angle. Collectively, these adaptations may contribute to a shift in the load–velocity relationship towards greater force production, particularly within the high-force region of the continuum [12].

Evidence supporting these adaptations was provided by Cormie et al. [14], who compared 10 weeks of heavy back-squat training (75–90% 1RM) with power training. Although both interventions improved jump performance, sprint ability and peak power, heavy resistance training produced greater improvements in maximal strength, lean body mass and muscle thickness, whereas power training elicited larger gains in movement velocity. These findings suggest that heavy resistance training enhances muscular power primarily by increasing force-producing capacity rather than contraction velocity.

Similar evidence of load-specific adaptations was reported by Moss et al., [13] who examined the effects of 9 weeks of maximal-effort resistance training at 15%, 35%, and 90% of 1RM. Training at 90% of 1RM produced the greatest improvements in maximal strength and demonstrated greater load specificity, whereas lower loads were more effective for improving power across lighter loading conditions. These findings further support the principle that heavy resistance training primarily enhances force-producing capacity, while the magnitude and specificity of power adaptations depend on the loading conditions employed.

Despite its effectiveness, heavy resistance training alone may be insufficient to maximize rapid force production performance because the relatively low movement velocities provide only a limited stimulus for velocity-specific adaptations [2,9]. Consequently, current evidence supports integrating heavy resistance training with higher-velocity methods, such as Olympic weightlifting, ballistic training, plyometric training and VBT, to optimize power development across the entire load–velocity continuum and maximize athletic performance [12].

#### 4.1.2. Ballistic Training

Ballistic training is the term used within strength and conditioning for resistance training methods designed to maximize power output by allowing continuous acceleration throughout the concentric phase of movement. Unlike traditional resistance exercises, which require a deceleration phase to resist the load, ballistic movements involve projecting either the external load or the athlete’s body, thereby maintaining high movement velocity until the end of the action [9,38]. It should be noted that ‘ballistic’ here has no mechanical definition, and should only be applied colloquially as a label for the types of movements fitting the description above.

Typical ballistic exercises include jump squats, bench throws and medicine-ball throws. Depending on the exercise, ballistic training may be performed using body mass alone or relatively light external loads. For example, jump squats are commonly performed with approximately 20–40% of 1RM, bench throws with approximately 30–45% of 1RM, whereas medicine-ball throws typically use appropriately selected ball masses according to the exercise and training objective [9,39]. This training method can promote higher concentric velocity, RFD and power output than conventional resistance training because acceleration is maintained throughout the movement [9,40].

Ballistic training may promote several neuromuscular adaptations associated with rapid force production, including improvements in motor-unit recruitment and firing behaviour, intermuscular coordination, and the effective use of the SSC, depending on the characteristics of the exercise and training stimulus. However, the relative contribution of neural and structural adaptations is likely influenced by factors such as external load, training volume, intervention duration, exercise selection, and participants’ training status. Although high-velocity ballistic training is often associated with adaptations that enhance rapid force production, structural adaptations, including changes in muscle morphology, may also occur depending on the characteristics of the training program [8,41].

Recent evidence further supports the relevance of ballistic resistance training for enhancing rapid force-production capabilities. In elite athletes, four weeks of maximum-intended-velocity ballistic training resulted in significant improvements in maximal propulsive power, RFD, and impulse, highlighting the potential importance of continuous acceleration and high contraction velocities for the development of rapid force characteristics [15].

Overall, ballistic training may be particularly appropriate when an adequate strength foundation has been established. When combined with heavy resistance training, it simultaneously develops force- and velocity-related qualities, providing a comprehensive stimulus for maximizing neuromuscular power and athletic performance [38,39].

#### 4.1.3. Olympic Weightlifting

Olympic weightlifting and its derivatives are widely recognized as effective resistance training methods for developing muscular power because they combine high force production with high movement velocity. Exercises such as the snatch, clean, hang clean and high pull require rapid force production and triple extension of the hip, knee and ankle, closely resembling the mechanical demands of sprinting, jumping and other rapid force production sporting actions [12].

These exercises produce some of the highest power outputs reported during resistance training, with peak power typically achieved using moderate-to-heavy loads (approximately 70–80% of 1RM). Their ability to simultaneously develop force and velocity may make Olympic weightlifting a useful approach for enhancing neuromuscular performance across a broad region of the load–velocity spectrum [9]. Adaptations are primarily neural, including improvements in motor-unit recruitment, firing frequency, intermuscular coordination and RFD [12].

Although Olympic weightlifting shares several characteristics with ballistic training, an important distinction exists. Ballistic exercises eliminate the deceleration phase by projecting the load or body, whereas Olympic lifts require the athlete to decelerate and receive the barbell during the catch phase [6]. Nevertheless, both methods maintain high concentric velocities and may contribute to the development of high-velocity force production, with Olympic lifts generally allowing greater power output under heavier loading conditions [8]. Moreover, weightlifting derivatives without the catch phase (e.g., high pulls or jump shrugs) may reduce technical demands while retaining the high-force and high-velocity characteristics that make Olympic weightlifting exercises effective for power development [42].

Recent evidence further supports their effectiveness. A systematic review by Cormier et al. [8] identified Olympic weightlifting and its derivatives as one of the most effective resistance training methodologies for improving mean power, peak power and mean propulsive power in team-sport athletes. Across interventions lasting 8–12 weeks, meaningful improvements were consistently reported using loads ranging from approximately 55 to 95% of 1RM. However, the review reported a low level of evidence supporting the effectiveness of Olympic weightlifting and its derivatives for improving lower-body power.

Consistent with these findings, Suchomel et al. [16] demonstrated that 10 weeks of weightlifting derivative training using catching derivatives, submaximal pulling or phase-specific loading improved CMJ force, velocity, and power, as well as isometric mid-thigh pull rapid force production and peak force. Notably, the submaximal pulling group demonstrated the greatest improvements in CMJ force, velocity, and power, whereas the phase-specific loading group showed the greatest increases in isometric mid-thigh pull rapid force production and peak force.

#### 4.1.4. Plyometric Training

Plyometric training is a neuromuscular method designed to enhance high-velocity power by exploiting the SSC, whereby a rapid eccentric action is followed immediately by a concentric action, thereby minimizing the duration of the amortization phase. This reduced amortization phase enhances the utilization of elastic energy stored by the rapid stretch of the muscle-tendon unit, allowing greater force production within short contraction times and improving rapid force production and athletic performance [10,12,43].

Within this broader classification, exercises may be further characterized according to SSC duration. Short SSC movements are typically characterized by contact times of approximately 100–250 ms, as observed in activities such as sprinting and certain high-intensity jumping tasks. In contrast, long SSC movements are characterized by longer movement durations (>250 ms), including exercises such as the countermovement jump (CMJ) and other jumping or throwing actions [9,44]. Performed primarily using body mass, these exercises closely replicate the demands of sprinting, jumping and rapid changes in direction. Their adaptations are predominantly neural, including improved motor-unit recruitment, firing frequency, intermuscular coordination and musculotendinous stiffness, which may contribute to improvements in RFD and reactive strength [12].

Recent evidence supports the effectiveness of plyometric training for improving selected physical performance outcomes. Sammoud et al. [43] reported significant improvements in 10, 20, and 30 m sprint performance, change-of-direction speed, and aerobic endurance following a 12-week plyometric training program in prepubertal male soccer players, whereas no significant changes were observed in 5 m sprint performance or maximal strength. These findings indicate that plyometric training may preferentially improve sprint and change-of-direction performance without necessarily producing significant gains in maximal strength.

Further supporting the effectiveness and specificity of plyometric training, a recent study reported that 8 weeks of plyometric training significantly improved peak power output and jump height across several unloaded and loaded jumping conditions, as well as 5, 10, and 20 m sprint performance [17].

Similarly, a network meta-analysis by Tan et al. [10], including 29 randomized controlled trials, found that the relative effectiveness of training methods varied according to the outcome assessed. Combined resistance and plyometric training showed the greatest probability of enhancing vertical jump performance, whereas OPL training ranked highest for sprint performance. The certainty of evidence for several comparisons involving jump training was rated as low. These findings suggest that the effectiveness of plyometric training may depend on the outcome assessed and on its integration with complementary resistance training methods.

Collectively, plyometric training may represent an important component of long-term power development. Although it does not maximize strength gains to the same extent as heavy resistance training, its capacity to improve reactive strength, movement velocity and SSC efficiency makes it an essential component of programs aimed at optimizing performance across the load–velocity continuum [10,12,43].

#### 4.1.5. Variable Resistance Training

VRT is a resistance training method in which the external load changes throughout the range of motion through the use of elastic bands, chains or other accommodating resistance devices combined with free weights. Unlike conventional resistance training, VRT progressively modifies the external resistance, reducing the limitations imposed by the sticking region and maintaining greater muscular tension throughout the movement [8].

By increasing resistance as mechanical advantage improves, VRT may allow force to be applied against a greater external load over a larger proportion of the concentric phase while potentially reducing the deceleration typically observed during conventional free-weight exercises [8]. However, the direct longitudinal evidence examining the effects of VRT on muscular power development remains comparatively limited.

Recent experimental evidence provides preliminary support for its potential application to lower-limb performance. In an 8-week randomized controlled intervention involving male college basketball players, chain-based VRT, consisting of a 55% 1RM barbell load combined with 10–30% 1RM from chains, produced significant within-group improvements in squat 1RM, countermovement jump, squat jump, reactive strength index, standing long jump, and 30 m sprint performance. However, no significant between-group differences were observed relative to the control group, limiting definitive conclusions regarding the superiority of VRT for power development [19].

These findings suggest that VRT may provide a useful complementary stimulus for improving strength and power-related performance, although the current evidence base remains insufficient to establish its comparative effectiveness relative to more extensively investigated training methods. Further longitudinal studies directly comparing VRT with conventional resistance and power-oriented training are required.

In practice, VRT typically combines conventional free weights with elastic bands or chains contributing approximately 10–30% of the total resistance. It can be applied to exercises such as squat, bench press and deadlift. Appropriate familiarization is recommended because accommodating resistance alters movement mechanics and requires technical proficiency [8,19].

#### 4.1.6. Isometric Training

Isometric training is a resistance training method in which force is produced without any observable change in muscle length or joint angle. Athletes generate force against an immovable object or maintain a fixed joint position, producing high levels of muscular tension despite the absence of movement. This characteristic allows the development of high levels of muscular tension despite the absence of movement, providing a stimulus for neuromuscular adaptations associated with strength and power development [8,45].

The primary adaptations to isometric training are predominantly neural, including increased motor-unit recruitment, voluntary activation, musculotendinous stiffness and improved neural drive [20,45].

A key characteristic of isometric training is its high degree of joint-angle specificity. Strength gains are greatest at or near the training angle; therefore, exercises should be prescribed at joint positions that closely replicate the biomechanical demands of the target movement. Common exercises include isometric squats, split-squat holds, bench-press holds, mid-thigh pulls and pushing or pulling against fixed bars or safety pins [8,45].

Current evidence indicates that isometric training effectively improves maximal strength, RFD and high-velocity performance. Consequently, isometric training should be considered a complementary method rather than a replacement for dynamic resistance training. The integration with heavy resistance training, Olympic weightlifting, ballistic training, plyometric training and velocity-based methods contributes to a more complete development of the load–velocity profile and athletic performance [8,45].

#### 4.1.7. Eccentric Overload Training

Eccentric overload training is a resistance training method that increases the mechanical stimulus during the eccentric phase of muscle contraction [8,46]. However, different approaches should not be considered mechanically equivalent. These may include accentuated eccentric loading, supramaximal eccentric actions, the use of weight releasers, and flywheel or inertial exercise, each of which produces eccentric loading through different mechanical mechanisms [22,46,47,48].

In addition to its chronic training adaptations, accentuated eccentric loading may also acutely enhance subsequent concentric performance. Recent evidence indicates that these acute effects are influenced by the magnitude of the subsequent concentric load. Specifically, a single supramaximal eccentric repetition at 120% 1RM enhanced subsequent eccentric and concentric velocity and power when followed by concentric repetitions at 65% 1RM, whereas similar improvements were not observed at 80% 1RM. These findings suggest that a sufficiently large differential between eccentric and concentric loading may be required to maximize the acute potentiating effects of accentuated eccentric loading [22].

The principal adaptations associated with eccentric overload training include improvements in eccentric strength, motor-unit recruitment, musculotendinous stiffness, and SSC efficiency, which may contribute to an enhanced ability to absorb braking forces and rapidly transition to concentric propulsion [8,47].

Collectively, eccentric overload training may be an effective strategy for improving eccentric strength, braking capacity and high-velocity performance [49]. Supporting this broader premise, Friedmann-Bette et al. [47] reported that 6 weeks of resistance training with eccentric overload resulted in significant improvements in squat jump performance, whereas conventional concentric–eccentric training did not elicit similar improvements. These changes were accompanied by alterations consistent with a faster muscle phenotype, suggesting that the adaptations induced by eccentric overload may extend beyond increases in maximal strength and muscle hypertrophy and contribute to enhanced high-intensity performance. Recent findings further suggest that neuromuscular adaptations to high-load eccentric training may occur independently of eccentric execution tempo [49]. More recently, an eight-week study directly comparing flywheel training and accentuated eccentric loading training in well-trained male sprinters reported significant improvements in lower-body strength, power, and sprint performance following both interventions. However, flywheel elicited greater improvements in countermovement jump performance and eccentric utilization ratio, suggesting a superior capacity to enhance elastic energy storage and utilization within the stretch-shortening cycle. These findings further emphasize that different eccentric overload modalities should not be considered mechanically or physiologically equivalent, as the specific characteristics of the eccentric stimulus may influence subsequent neuromuscular adaptations [23].

When integrated with traditional resistance and high-velocity training methods, it provides a unique stimulus for athletes involved in sports characterized by repeated accelerations, decelerations and rapid changes in direction [7,8,47].

### 4.2. Prescription and Monitoring Strategies

#### 4.2.1. Optimal Power Load

OPL training is a resistance training method in which the external load is selected to maximize mechanical power output rather than being prescribed as a fixed percentage of 1RM. Based on the load–velocity relationship, OPL seeks the load at which the optimal combination of force and movement velocity is achieved, thereby maximizing power production [25,39].

Power output is commonly monitored using variables such as mean power, peak power and mean propulsive power, the latter being considered particularly useful because it quantifies power exclusively during the propulsive phase of the movement, excluding the braking phase [8,25]. The increasing availability of linear position transducers and other velocity-monitoring devices has facilitated the individual identification of OPL, allowing practitioners to prescribe training according to each athlete’s mechanical profile.

Unlike traditional percentage-based loading, OPL is highly individualized and varies according to the exercise and athlete. According to the studies reviewed by Cormier et al. [8], optimal power was achieved with loads ranging from approximately 30% to 80% of 1RM, although ballistic exercises typically required lighter loads than traditional resistance exercises. Some studies prescribed the load corresponding to maximal power output, whereas others used velocity thresholds to maintain athletes within the optimal power zone.

Training at OPL may simultaneously target force- and velocity-related qualities by enhancing motor-unit recruitment, intermuscular coordination and rate of force development while maintaining sufficient mechanical loading to stimulate muscular adaptations. Consequently, OPL occupies a variable, exercise-dependent position along the load–velocity spectrum, generally representing a bridge between heavy-resistance and ballistic loading conditions.

Therefore, OPL is an evidence-based and highly individualized strategy for enhancing high-velocity performance. Rather than replacing maximal resistance training, it may be integrated with heavy resistance, ballistic and velocity-based methods to optimize power development across the entire load–velocity continuum [8,25].

#### 4.2.2. Velocity-Based Training

VBT is a resistance training method in which movement velocity is used to prescribe, monitor and adjust training intensity in real time. Unlike traditional percentage-based approaches, VBT uses movement velocity as an objective measure of exercise performance and can support the daily autoregulation of training loads by accounting for changes in performance capacity and training readiness [50].

The method is based on the inverse load–velocity relationship, whereby movement velocity decreases as external load increases. Because concentric velocity is closely related to relative exercise intensity, monitoring barbell velocity enables practitioners to prescribe training according to movement quality rather than absolute load, reducing the need for frequent 1RM testing. Linear position transducers and other velocity-monitoring devices provide immediate feedback, allowing coaches to adjust external loads and maintain the desired training stimulus throughout the session [8,50].

An important feature of VBT is the use of velocity-loss thresholds to regulate training volume and manage within-session performance decrements. Rather than completing a predetermined number of repetitions, sets can be terminated once movement velocity reaches a predefined threshold. Different velocity-loss thresholds may result in distinct physiological and performance adaptations; however, these responses appear to depend on factors such as the population, exercise, training volume, intervention duration and the specific threshold applied [8].

VBT is also closely integrated with OPL training by enabling practitioners to identify and maintain the velocity associated with maximal power output. This combination may facilitate individualized training while supporting movement quality and minimizing unnecessary fatigue, particularly during competitive periods [25].

According to the systematic review by Cormier et al. [8], VBT is an effective strategy for improving lower-body power in team-sport athletes, with interventions lasting 7–10 weeks consistently demonstrating favourable improvements in high-velocity performance. Although the evidence remains less extensive than for traditional resistance or combined training, VBT offers greater precision, individualization and flexibility in load prescription. Similarly, VBT may also represent an interesting training method in individual sports. However, in combat sports athletes, Chen et al. [26] demonstrated that both VBT and percentage based resistance training improved measures of upper-body strength and general and sport-specific power after an 8-week intervention, with percentage based resistance training producing greater improvements in several power-related outcomes.

Overall, VBT represents a potentially useful approach to prescribing resistance training. By continuously monitoring movement velocity, it enables practitioners to autoregulate training intensity, support individualized power development and facilitate athlete monitoring, making it a potentially valuable component of modern strength and conditioning programs [8,38,50].

### 4.3. Set Configurations and Exercise Sequencing

#### 4.3.1. Cluster Sets

Cluster set training is a resistance training method in which brief rest intervals are inserted within a set, dividing the prescribed repetitions into smaller clusters. Unlike traditional set configurations, where repetitions are performed continuously, cluster sets allow partial recovery during the set, helping to maintain movement intent and therefore velocity, force production and power output while reducing neuromuscular fatigue [18,51,52]. For example, a traditional configuration of 4 × 4 repetitions may be compared with a cluster configuration of 4 × (2 + 2) repetitions separated by 20 s of intra-set rest. In both cases, the total training volume (16 repetitions) and the inter-set rest interval (e.g., 3 min) are matched, with the only difference being the brief intra-set recovery period. This configuration helps maintain movement velocity and power output throughout the training session.

The physiological rationale for cluster sets is based on the partial restoration of phosphocreatine stores and the attenuation of metabolite accumulation during intra-set recovery periods. Consequently, athletes are able to sustain higher concentric velocity, peak force and power throughout the set, increasing the number of high-quality repetitions performed while reducing the decline in mechanical performance typically observed during traditional resistance training [52].

Supporting this rationale, Hardee et al. [18] demonstrated that cluster set configurations can reduce power loss and perceived exertion, while García-Ramos et al. [51] showed that cluster set configurations allow athletes to sustain higher movement velocities across a greater number of repetitions, thereby attenuating velocity loss and fatigue within the set.

From a practical perspective, cluster sets may be particularly beneficial when the primary objective is to maximize power output while minimizing fatigue. Short intra-set rest intervals (typically 10–30 s) enable athletes to maintain movement quality during moderate-to-high intensity resistance training, potentially making this method suitable for sports requiring repeated high-velocity actions, such as sprinting, jumping, Olympic weightlifting and combat sports.

#### 4.3.2. Complex-Contrast Training

Complex-contrast training is a strength–power training method that utilizes two exercises with different loads and movement velocities, organized in a set-by-set sequence within the same training session. By combining high-force and high-velocity exercises, this method simultaneously targets different regions of the load–velocity continuum, promoting improvements in muscular power and athletic performance [53].

Although complex-contrast training has traditionally been described as pairing heavy-resistance and high velocity exercises within the same training session, the scientific literature recognizes several organizational variants of this method. These include complex-descending, in which all heavy-resistance sets are completed before the high velocity exercise sets; complex-ascending, where high velocity exercises precede heavy-resistance exercises; complex-contrast, in which heavy and high velocity exercises are alternated on a set-by-set basis; and French contrast training, a more advanced variation that combines heavy, ballistic, loaded ballistic, and assisted plyometric exercises within the same sequence. Although all of these approaches are designed to enhance neuromuscular performance and power development, the complex-contrast configuration has received the greatest scientific attention [53].

Typically, a heavy strength exercise (e.g., back squat) is immediately followed by a plyometric exercise (e.g., bounds or hops), aiming to exploit the phenomenon of post-activation performance enhancement (PAPE). To maximize transfer, the paired exercises should be biomechanically similar, with appropriate management of training load and recovery to optimize potentiation while limiting fatigue [54].

Although PAPE provides the acute physiological rationale for pairing heavy and high-velocity exercises within the same session, improvements following multi-week complex-contrast training are attributed to chronic neuromuscular adaptations induced by repeated training exposure, rather than the acute potentiation response itself [10,54].

Recent longitudinal evidence further supports the effectiveness of complex training for enhancing power-related performance. Wang et al. [27] demonstrated that an 8-week complex training intervention improved CMJ performance and produced greater improvements than resistance training alone in female basketball players. Similarly, Xu et al. [28] reported that 24 weeks of complex training improved jump performance and eccentric-strength-related variables in national-level track and field athletes.

Therefore, complex-contrast training may represent an effective strategy for improving high-velocity performance by combining the complementary adaptations of heavy resistance and high-velocity exercise. When appropriately integrated within a periodized strength and conditioning program, it may provide a complementary stimulus for enhancing power across the load–velocity spectrum [10,53].

### 4.4. Periodization Models

#### Triphasic Training

Triphasic training is a resistance training method originally proposed by Dietz and Peterson [55] that systematically organizes training around the three phases of muscle action: eccentric, isometric, and concentric. Unlike traditional resistance training approaches that may address these phases simultaneously, triphasic training dedicates specific training blocks to emphasizing each phase. The model has been proposed as a structured approach for developing and integrating different strength- and power-related qualities across successive training phases.

Within the triphasic training framework, the eccentric phase is proposed to emphasize eccentric force production and tolerance to high mechanical loads through controlled eccentric actions performed with moderate-to-high external loads. The subsequent isometric phase emphasizes force production and force transmission at specific joint positions, whereas the concentric phase prioritizes a high intent to move rapidly. These proposed emphases are intended to sequentially target different mechanical characteristics of muscle actions [55].

From a load–velocity perspective, triphasic training cannot be positioned within a single region of the continuum because its sequential structure targets multiple mechanical qualities over time. Consequently, the model is intended to address multiple regions of the load–velocity continuum over successive training phases by integrating force- and velocity-oriented training emphases.

Preliminary evidence supporting this approach remains limited. For example, Rebelo et al. [56] examined a six-week preseason triphasic training program in elite male volleyball players and reported improvements in lower-body maximal strength (back squat), upper-body maximal strength (bench press), and squat jump performance. Back squat, bench press, and squat jump performance improved by 18.43% (d = 0.71), 7.99% (d = 0.45), and 3.16% (d = 0.27), respectively. However, these findings should be interpreted with caution because the study had no control group and a relatively small sample size.

Similarly, Kaya and Pınar [29] investigated the effects of a six-week triphasic training program in 16 male athletes with previous resistance training experience. Participants completed sequential eccentric, isometric, and concentric training phases while performing the program twice weekly. The study additionally compared two intra-set rest configurations using cluster sets, with either 15 or 30 s of rest between repetitions. Both groups demonstrated significant improvements in countermovement jump height and reactive strength index following the intervention, with the 30 s cluster set configuration showing greater pre-to-post improvements than the 15 s configuration. These findings suggest that a triphasic training program may be associated with improvements in high-velocity force production and reactive performance, while also indicating that the configuration of intra-set rest intervals may influence the magnitude of adaptation. However, because both groups completed the triphasic training program and no non-triphasic or control condition was included, the independent contribution of the triphasic training model cannot be isolated from the effects of the overall resistance training intervention and cluster set configuration [29].

Overall, triphasic training may represent a practitioner-derived periodization model that proposes the sequential emphasis of eccentric, isometric, and concentric actions within strength and power development. The currently available longitudinal evidence provides preliminary support for potential improvements in strength, jump, and reactive performance; however, the number of studies remains limited, and existing interventions do not yet allow the independent effects of the triphasic structure to be clearly distinguished from other training variables, including exercise selection, loading, and set configuration. Although its theoretical rationale may provide a useful framework for organizing training, the direct empirical evidence supporting its effectiveness and the optimal prescription of its individual phases remains limited. Therefore, its practical application should currently be considered evidence-informed rather than empirically validated and should be individualized according to the athlete and training context [29,55,56].

The loading, repetition, rest, and velocity characteristics presented in Table 2 should be interpreted as general examples of possible implementation rather than universal prescription thresholds, as these variables are exercise-, population-, and measurement-dependent [2,8,9].

## 5. Practical Applications

### 5.1. Practical Considerations for Power Training

While understanding the physiological mechanisms underpinning muscular power is essential, the effective implementation of power-oriented resistance training ultimately depends on appropriate exercise selection, periodization, and the strategic combination of training methods. Importantly, the practical recommendations presented in this review are primarily derived from evidence obtained in healthy, physically active adults and athletic populations and should not be generalized uniformly to youth, older adults, or clinical populations. Loading ranges, execution velocities, and training-block durations should therefore be individualized according to the athlete’s characteristics, including training status, sex, sport-specific demands, competitive context, technical proficiency, injury history, and the available monitoring tools.

Because each resistance training method targets specific regions of the load–velocity continuum and elicits distinct neuromuscular adaptations, no single approach is universally superior. Instead, practitioners may consider integrating multiple evidence-based methods according to the athlete’s training status, sport-specific demands, competitive calendar, and performance objectives.

Where available, practitioners should consider not only whether a training intervention produces statistically significant improvements but also the magnitude and certainty of the observed effects. Meta-analyses and systematic reviews provide useful information regarding the magnitude of training-induced adaptations; however, direct quantitative comparisons across all power-oriented training methods remain limited because studies differ substantially in participant characteristics, exercise selection, intervention duration, training prescription, and outcome measures. Consequently, comparisons between methods should be interpreted cautiously and should integrate quantitative findings from method-specific meta-analyses with the mechanical characteristics and practical requirements of the training intervention [71].

Training dose is another important consideration when translating the available evidence into practice. Current evidence indicates that the magnitude of adaptation to resistance training is influenced by variables such as training intensity, intervention duration, and the outcome being targeted [71]. However, a universal minimum effective dose for muscular power development across all training methods has not been established. Available minimum-dose research has primarily focused on maximal strength rather than muscular power and demonstrates that the minimum effective dose may vary according to the population and outcome assessed [72]. Therefore, the volume, frequency, intensity, and duration of the methods presented in this review should not be interpreted as universally optimal or minimum effective prescriptions unless directly supported by method-specific evidence.

Similarly, although maximal strength is an important contributor to muscular power and athletic performance, the available literature does not support a single universal strength threshold at which athletes should transition from predominantly strength-oriented to predominantly velocity-oriented training. Stone et al. [1] emphasized the importance of maximal strength for the expression of power and sport-related performance, but the magnitude of this relationship depends on the specific task and athlete characteristics. Accordingly, decisions regarding the relative emphasis placed on maximal-strength and velocity-oriented training should be based on the athlete’s individual performance profile, training history, and sport-specific demands rather than on a single absolute strength criterion.

Effective power development requires balancing force production and movement velocity while simultaneously managing fatigue, technical complexity, and injury risk. Consequently, methods emphasizing maximal force production may be prioritized during periods of lower competition density, whereas strategies that preserve movement quality and minimize fatigue may be more appropriate during the competitive season [73].

Another important consideration is exercise selection. Although the mechanical characteristics of each training method determine the primary training stimulus, choosing exercises that closely replicate the movement patterns, force application, and velocity demands of the target sport may further enhance transfer to athletic performance [32].

Finally, combining complementary training strategies throughout the season allows practitioners to exploit the unique advantages of each method while minimizing their individual limitations.

### 5.2. Representative Exercises for Power-Oriented Resistance Training Methods

Exercise selection represents one of the most important practical decisions when designing power-oriented resistance training programs. Although the physiological adaptations associated with each training method are primarily determined by the mechanical stimulus imposed during training, selecting exercises that reflect the biomechanical characteristics of the target sport may substantially improve the transfer of training adaptations to athletic performance. Consequently, practitioners should prioritize exercises that simultaneously develop force production, movement velocity, coordination, and movement specificity.

For most athletes, multi-joint exercises involving large muscle groups are recommended because they closely resemble the mechanical demands of most sports. However, exercise selection should also consider the athlete’s technical competency, training experience, injury history, available equipment, and the specific objectives of the training phase. Highly technical exercises, such as Olympic weightlifting derivatives, require greater coaching expertise and movement proficiency, whereas simpler exercises (e.g., jumps and throws) may provide similar neural stimuli with lower technical demands (Table 3).

Although these exercises are among the most frequently implemented in both scientific research and applied strength and conditioning practice, they should not be considered universally applicable. Instead, exercise selection should be individualized according to the athlete’s technical proficiency, sport-specific movement patterns, injury profile, and available training resources.

### 5.3. Periodization of Resistance Training Methods Using a Block Periodization Approach

The following block-periodization framework represents an evidence-informed practical model that integrates established principles of block periodization with the physiological and mechanical characteristics of different power-oriented training methods. Although individual components of this framework are supported by the available literature, the specific combination, sequencing, and duration of the methods proposed below have not been prospectively validated as an optimal training sequence. The proposed durations should be interpreted as approximate guidelines and adapted according to the athlete’s training status, competition calendar, sport-specific demands, and individual response to training. Therefore, the recommendations should be interpreted as an integrative practical synthesis rather than as prescriptive evidence-based guidelines.

The effectiveness of power-oriented resistance training depends not only on selecting appropriate training methods but also on their strategic sequencing throughout the long-term training process [74,75]. Block periodization has been proposed as an organizational approach in which training is structured into relatively concentrated blocks emphasizing specific physical qualities, with the aim of facilitating the sequential development of targeted adaptations [76]. However, although the theoretical rationale and practical application of block periodization are well described, the superiority of this approach over alternative periodization strategies has not been consistently established across athletic populations [77,78]. Since each resistance training method induces distinct neuromuscular adaptations and imposes different levels of mechanical stress, practitioners may organize training interventions according to the objectives of each training block.

The following block-periodization framework should be interpreted as an evidence-informed practical model rather than as a prospectively validated optimal sequence. Its purpose is to provide a rational framework for integrating the training methods discussed throughout this review according to their primary mechanical and physiological objectives. Although the individual methods and periodization principles are supported to varying degrees by the available literature, the specific sequencing and duration of the proposed blocks have not been directly tested as a single integrated model. Therefore, the suggested timeframes should be considered approximate and adapted according to the athlete’s training status, sporting demands, competitive calendar, training history, and individual response.

For the purposes of the present practical framework, an initial General Capacity phase may precede the more concentrated training blocks. This phase is not a formal component of Issurin’s model but is included here as an additional preparatory period intended to establish general physical preparedness and facilitate the subsequent implementation of more specialized training.

The General Capacity phase, typically lasting 2–4 weeks, may provide a foundation for subsequent training by emphasizing general physical preparedness, movement quality, and overall work capacity. During this phase, training should primarily include general resistance training, concurrent training, and circuit-based resistance exercise performed at relatively low-to-moderate intensities to establish a robust physical base while minimizing excessive mechanical stress [38].

During the Accumulation phase, which generally lasts 3–4 weeks, the primary objective is to establish a robust foundation of maximal strength and general neuromuscular capacity that can subsequently be transferred into sport-specific power. Accordingly, training should emphasize methods that promote structural and neural adaptations, including heavy resistance training, accentuated eccentric loading, variable resistance training, and isometric training. Training volume is generally higher during this phase, whereas intensity progressively increases to develop force-production capacity and prepare athletes for subsequent specialized training blocks [38,76].

As athletes progress into the Transmutation phase, typically lasting 2–4 weeks, the emphasis shifts from maximizing force production toward converting maximal strength into high-velocity force production and muscular power [38,76]. During this phase, practitioners may progressively integrate ballistic training, plyometric training, Olympic weightlifting derivatives, OPL, complex-contrast training, and cluster training, while maintaining an adequate stimulus of heavy resistance training to support the maintenance of maximal strength and the transfer of force across the load–velocity continuum.

The Realization phase, which usually lasts approximately 2 weeks, aims to maximize sport-specific performance while minimizing accumulated fatigue before competition. Consequently, overall training volume should be reduced, whereas movement quality, execution velocity, and exercise specificity become the primary priorities. VBT, OPL, ballistic training, plyometric training, complex-contrast training, cluster training, and selected Olympic weightlifting derivatives may be particularly appropriate during this phase because they emphasize high movement velocity and neuromuscular performance. Heavy resistance training may still be incorporated at a reduced volume to maintain maximal strength without compromising competitive performance [38,74,76].

Following periods of intensified training or competition, an additional Restoration phase may be incorporated within the present practical framework to facilitate physiological recovery, reduce accumulated fatigue, and restore readiness before initiating a subsequent training cycle. This phase is not presented as a formal component of Issurin’s model but rather as a practical recovery-oriented period included to accommodate the demands of long-term training and competition. During this phase, practitioners may emphasize low-load resistance training, circuit training, concurrent training, mobility exercises, and general conditioning, aiming to preserve neuromuscular adaptations while promoting recovery through reduced mechanical stress [38].

The block periodization provides a logical framework for integrating resistance training methods according to their physiological objectives. Rather than considering these methods as isolated interventions, practitioners may strategically sequence them throughout successive training blocks to support long-term adaptations and power development while considering sport-specific performance demands, fatigue, and injury risk. These methods should therefore be viewed as complementary components of an integrated long-term training method rather than as isolated training interventions [76].

## 6. Limitations

Despite providing a comprehensive overview of current power training methods, this narrative review has several limitations that should be acknowledged. First, although a structured database search and independent screening process were used for the evidence-mapping component, the review was not designed or reported as a formal systematic or scoping review. Therefore, the identification and contextualization of literature may be susceptible to selection bias. Second, the included studies were highly heterogeneous regarding participant characteristics, sporting disciplines, training status, intervention protocols, and outcome measures, limiting direct comparisons across training methods. Furthermore, relatively few studies have directly compared different power training methods under standardized conditions, and many practical recommendations are derived from indirect performance outcomes (e.g., jump height or sprint performance) rather than direct assessments of load–velocity characteristics. Additionally, youth populations and older adults remain underrepresented in the available literature, restricting the generalizability of current findings. The evidence-mapping component also did not include a formal study-level risk-of-bias or methodological quality assessment, which should be considered when interpreting the findings. Finally, although the proposed periodization framework is grounded in the available evidence and established training principles, it should be interpreted as an evidence-informed practical model rather than a fully validated sequence, highlighting the need for future longitudinal and comparative research. Only articles published in English were considered, which may have resulted in the exclusion of relevant evidence published in other languages.

## 7. Conclusions

Muscular power is a fundamental determinant of athletic performance and represents a complex physical quality influenced by the interaction between force and velocity. Consequently, optimizing muscular power may benefit from a multifaceted approach that considers the specific physiological adaptations elicited by different resistance training methods rather than relying on a single training strategy.

Based on the available evidence, heavy resistance training appears to represent an important method for developing maximal force capacity, whereas ballistic training, Olympic weightlifting, and plyometric training show consistent evidence of effectiveness for enhancing high-velocity force production and related power outcomes. VBT and OPL may provide useful individualized approaches for prescribing training intensity and optimizing power output, while cluster sets may help maintain movement quality and manage fatigue during high-intensity sessions. Variable resistance, isometric training, and eccentric overload training appear to provide complementary neuromuscular stimuli under specific training conditions, although the quantity and strength of evidence supporting these approaches vary. Emerging approaches, such as complex-contrast and triphasic training, show promising results; however, current evidence remains comparatively limited and further high-quality comparative studies are required before definitive recommendations can be made. Accordingly, the integration of these methods within a periodized program should be considered an evidence-informed practical approach rather than a universally applicable prescription.

From a practical perspective, effective power development extends beyond selecting appropriate exercises or training loads. Exercise selection, periodization, and the combination of complementary training methods should be individualized and informed by the available evidence, athlete characteristics, and contextual demands. Furthermore, training programs should remain individualized, recognizing that no single method is universally optimal across all sports, competitive levels, or athlete populations.

Despite the substantial body of evidence supporting resistance training interventions for muscular power development, several important gaps remain. Future research should directly compare combinations of resistance training methods, investigate season-long periodized interventions performed alongside regular sport-specific training, and establish evidence-based guidelines for integrating multiple training strategies throughout the competitive season. Greater emphasis should also be placed on athlete adherence, the effects of congested competition schedules, and the concurrent monitoring of performance and fatigue, including neuromuscular and cognitive or mental fatigue, as these factors may collectively influence readiness and the expression of high-velocity force and power to optimize training prescription and recovery. Finally, future studies should determine the optimal prescription variables for athletes from different sports, competitive levels, sexes, and age groups through ecologically valid interventions conducted under real-world training conditions.

## Figures and Tables

**Table 1:** Study designs, participant characteristics, intervention characteristics, training methods, and main findings of studies examining muscular power development included in the evidence mapping.

Authors	Study Design	Participant Characteristics (*n*; Sex; Sport; Age)	Intervention Characteristics (Duration; Outcome Measures)	Training Method	Main Findings
Moss et al. (1997) [13]	Controlled experimental.	*n* = 30; men; physically active; 22.7 ± 1.4, 24.0 ± 3.4, and 22.9 ± 2.8 y.	Duration: 9 weeks; Outcome measures: Elbow flexor 1RM, PP, velocity, muscle cross-sectional area, and load–power/load–velocity relationships.	Heavy resistance training.	Power increased across all groups, with load-specific adaptations: heavier loads produced greater improvements in power at higher loads in the single-joint elbow flexor exercise. Strength increased by 15.2%, 10.1%, and 6.6% in the 90%, 35%, and 15% 1RM groups, respectively; PP was greatest at 35–50% 1RM.
Cormie et al. (2010) [14]	RCT.	*n* = 24; men; relatively weak men; 23.9 ± 4.8 y.	Duration: 10 weeks; Outcome measures: 1RM, jump performance/PP, 40 m sprint performance, force–velocity characteristics, jumping mechanics, muscle architecture, and neural drive.	Heavy resistance training; Ballistic.	Both heavy resistance and ballistic training improved PP (17.7 ± 9.3% and 17.6 ± 4.5%, respectively; η^2^ = 0.357 and 0.300) and 40 m sprint performance (2.2 ± 1.9% and 3.6 ± 2.3%, respectively), with no significant between-group differences. Heavy resistance training produced a greater increase in squat 1RM than ballistic training (31.2 ± 11.3% vs. 4.5 ± 7.1%; η^2^ = 0.420). Overall, athletic performance improvements were similar in these relatively weak individuals after 10 weeks of training.
Lecce et al. (2025) [15]	RCT.	*n* = 20; men and female; track and field sprinters and weightlifters; G1: 24.7 ± 3.3 y, G2: 23.0 ± 3.3 y.	Duration: 4 weeks; Outcome measures: 1RM, MaxPP, RFD, impulse.	Ballistic.	Maximal-intended-velocity training produced greater improvements in absolute MaxPP (+27%, WG d = 2.46), RFD (+29%, WG d = 2.49), and impulse (+21%, WG d = 2.08) than moderate-velocity training (+9%, WG d = 1.13; +8%, WG d = 0.96; and +8%, WG d = 1.30, respectively). When normalized to 1RM, maximal-intended-velocity training increased MaxPP (+13 W·%1RM^−1^, WG d = 1.41), RFD (+61%1RM·s^−1^, WG d = 1.23), and impulse (+5%1RM·s, WG d = 1.00), whereas moderate-velocity training decreased MaxPP (−3 W·%1RM^−1^, WG d = 0.74) and RFD (−15%1RM·s^−1^, WG d = 0.85), with no significant change in impulse.
Suchomel et al. (2022) [16]	Randomized three-group RM.	*n* = 27; men; NCAA Division III athletes; CATCH: 22.8 ± 3.6 y, PULL: 22.2 ± 2.3 y, OL: 22.3 ± 1.2 y.	Duration: 10 weeks; Outcome measures: Muscle architecture, CMJ force, velocity and PP, IMTP peak force and rapid force production (force at 100–250 ms).	Olympic weightlifting.	10 weeks of submaximal pulling or phase-specific loading weightlifting derivatives improved CMJ force, velocity, and power, as well as IMTP rapid force production and peak force. The PULL group showed the greatest improvements in CMJ velocity (WG g = 0.71) and power (WG g = 0.54), whereas the OL group demonstrated the greatest increases in IMTP force at 100 ms (WG g = 0.64) and peak force (WG g = 0.73). The CATCH group showed trivial effects on CMJ variables and trivial-to-small decreases in IMTP performance.
Berton et al. (2022) [17]	RCT.	*n* = 45; men; physically active; G1: 24.1 ± 4.2 y, G2: 23.9 ± 4.7 y, CG: 24.3 ± 3.2 y.	Duration: 8 weeks; Outcome measures: PP output and jump height in the SJ, CMJ, and loaded CMJ (60% and 80% body mass), and sprint speed over 5, 10, 20, and 30 m.	Plyometric training; Olympic weightlifting.	Plyometric training significantly improved PP output and jump height in the SJ, PP output in the CMJ, and PP output and jump height in the loaded CMJ at 60% body mass. Compared with weightlifting, plyometric training showed greater improvements in SJ PP (BG ES = −1.21, 95% CI −2.04 to −0.44), SJ jump height (BG ES = −0.79, 95% CI −1.56 to −0.04), and CMJ PP (BG ES = −0.93, 95% CI −1.71 to −0.19). Sprint performance also improved over 5, 10, and 20 m, but not 30 m.
Hardee et al. (2012) [18]	Randomized RM experimental.	*n* = 10 men; recreational weightlifters; 23.6 ± 0.37 y.	Duration: Acute (three experimental sessions over 2 weeks); Outcome measures: PP and RPE.	Cluster; Olympic weightlifting.	Increasing IRR reduced power loss and perceived exertion during repeated power cleans at 80% 1RM. PP decreased by 9.0%, 3.0%, and 2.1% with 0, 20, and 40 s IRR, respectively, while RPE decreased from 7.43 to 6.46 and 5.30. The 40 s IRR condition resulted in significantly lower fatigue and RPE than the shorter IRR conditions.
Peng et al. (2025) [19]	RCT.	*n* = 27; men; college basketball players; G1: 19.3 ± 0.8 y, G2: 19.9 ± 1.1 y, CG: 19.8 ± 1.1 y.	Duration: 8 weeks; Outcome measures: Squat 1RM, CMJ height, SJ height, DJ-RSI, SLJ distance, 30 m sprint time, and left and right thigh circumference.	Variable resistance training.	Eight weeks of VRT significantly improved squat 1RM (+9.32%, WG ES = 0.94), CMJ (+5.00%, WG ES = 0.66), SJ (+4.96%, WG ES = 0.39), DJ-RSI (+12.14%, WG ES = 0.85), SLJ (+7.88%, WG ES = 1.21), and 30 m sprint performance (−6.61%, WG ES = 1.32). No significant changes were observed in the CG, and no significant between-group differences were found between VRT and blood flow restriction training combined with VRT.
Tsoukos et al. (2016) [20]	Randomized and counterbalanced RM.	*n* = 14; men; national-level track and field power athletes; 27.1 ± 7.0 y.	Duration: Acute (three experimental trials conducted 1 week apart); Outcome measures: CMJ height, peak force, average force, total impulse, and muscle fatigue.	Isometric training.	Maximal isometric squats at 140° increased CMJ height by 3.8 ± 1.2% at 12 min post-exercise, whereas no significant change occurred at 90°. Among high jumpers, CMJ increased by 5.4 ± 1.4% (d = 0.99) and 7.4 ± 1.2% (d = 1.60) following the 90° and 140° conditions, respectively, with a greater response at 140° (d = 0.62).
Lum et al. (2022) [21]	RCT.	*n* = 22; 16 men, 6 female; endurance runners; 37 ± 6 y.	Duration: 6 weeks; Outcome measures: CMJ height, peak force, PP, time to take-off, unweighting, braking and propulsion phase duration, reactive strength index modified, IMTP peak force, relative peak force, and force at 100, 150, and 200 ms.	Isometric training; Plyometric training.	Six weeks of plyometric and isometric training similarly improved CMJ height (WG d = 0.48 and 0.47, respectively). Plyometric training also improved CMJ PP (WG d = 0.31), whereas isometric training increased countermovement depth (WG d = 1.02), propulsive phase duration (d = 0.65), IMTP peak force (WG d = 0.55), and relative IMTP peak force (WG d = 0.87). Between-group effects favored isometric training for countermovement depth (d = 0.96) and relative IMTP peak force (d = 0.90). Neither training method improved RFD.
Merrigan et al. (2021) [22]	Randomized counterbalanced crossover.	*n* = 21; men; physically active; 24.0 ± 4.2 y.	Duration: Acute; Outcome measures: Concentric and eccentric mean and peak velocity, force, and power during the back squat.	Eccentric overload training.	A single supramaximal eccentric repetition at 120% 1RM enhanced subsequent concentric velocity and power during back-squat repetitions performed at 65% 1RM, with significant protocol × repetition effects for concentric mean power (ηp^2^ = 0.12) and eccentric PP (ηp^2^ = 0.16). Pairwise comparisons showed moderate-to-large effects (d = 0.6–2.0) favoring AELT across selected repetitions. These effects were less consistent at 80% 1RM, highlighting the importance of the eccentric–concentric load differential.
Hu et al. (2024) [23]	Randomized two-group RM.	*n* = 14 (FWT = 7; AELT = 7); men; elite academy sprinters; 23.5 ± 0.5 y.	Duration: 8 weeks; Outcome measures: Back squat 1RM, CMJ height, SJ height, EUR, SLJ distance, 30 m sprint time, mean and peak concentric velocity and power, and velocity and power decline and maintenance.	Eccentric overload training	Both FWT and AELT significantly improved lower-body strength, jump performance, and 30 m sprint performance. FWT increased CMJ height (+13.3%, WG d = 5.42), SJ height (+6.0%, WG d = 2.94), and EUR (+6.5%, WG d = 4.42), whereas AELT increased CMJ height (+7.4%, WG d = 3.44) and SJ height (+6.4%, WG d = 2.21), but not EUR (+0.9%, WG d = 0.63; *p* = 0.199). Improvements in CMJ height and EUR were significantly greater with FWT than AELT (*p* < 0.001).
Freitas et al. (2019) [24]	Quasi-experimental pre–post design with randomized group allocation.	*n* = 18; men; semi-professional basketball players; 21.3 ± 4.3 y.	Duration: 6 weeks; Outcome measures: 1RM (half-squat, bench press, and hip thrust), CMJ height and PP, SLJ distance, 10 m sprint time, and COD performance.	Optimal power loading.	Both OPL and MCT produced moderate-to-large improvements in lower-body strength, with half-squat 1RM increasing by 10.8% (WG ES = 0.64) and 17.2% (WG ES = 0.64), respectively, and hip-thrust 1RM by 23.5% (WG ES = 0.98) and 28.2% (WG ES = 1.23), respectively. However, both protocols produced only trivial effects on CMJ height (WG ES = 0.17 and 0.15) and CMJ PP (WG ES = 0.16 and 0.22), for OPL and MCT, respectively. OPL produced a small effect on 10 m sprint performance (WG ES = 0.29) and COD (WG ES = 0.42), whereas MCT produced a small effect on SLJ (WG ES = 0.27) and a moderate effect on COD (WG ES = 0.75).
Rauch et al. (2018) [25]	Randomized parallel-group RM.	*n* =15; female; collegiate volleyball players; 19.3 ± 1.4 y.	Duration: 7 weeks; Outcome measures: CMJ and SJ height, T-test agility, 1RM and PP output (back squat, bench press and deadlift).	Velocity-based training; Optimal power loading.	Within the OPL group, back-squat, bench-press, and deadlift 1RM increased by 18.3% (ES = 1.57), 10.2% (ES = 0.72), and 22.9% (ES = 1.49), respectively, while PP increased by 19.8% (ES = 0.79), 27.9% (ES = 1.68), and 20.1% (ES = 1.77), respectively. Within the VBT group, back-squat, bench-press, and deadlift 1RM increased by 19.6% (ES = 1.72), 8.5% (ES = 0.58), and 10.9% (ES = 0.88), respectively, while PP increased by 18.3% (ES = 0.86), 14.5% (ES = 0.81), and 15.7% (ES = 1.32), respectively. OPL produced a greater improvement in deadlift 1RM than VBT (*p* ≤ 0.05).
Chen et al. (2025) [26]	Randomized parallel-group controlled trial.	*n* = 24; men; combat sports athletes; 21.5 ± 2.1y.	Duration: 8 weeks; Outcome measures: Bench press 1RM, average velocity, average power and distance in 4- and 6 kg medicine ball throws, and average power in left and right Seoi-nage.	Velocity-based training.	Both VBT and PBRT significantly improved upper-body strength and power. However, PBRT produced greater improvements in 4 kg medicine-ball velocity (ηp^2^ = 0.527), power (ηp^2^ = 0.256), and throw distance (ηp^2^ = 0.222), as well as left (ηp^2^ = 0.449) and right (ηp^2^ = 0.228) Seoi-nage power. No significant between-group difference was observed for bench-press 1RM (ηp^2^ = 0.102).
Wang et al. (2024) [27]	RCT.	*n* = 32; female; college basketball players; CT: 19.7 ± 3.9 y; TRT: 19.9 ± 2.1 y	Duration: 8 weeks; Outcome measures: 5-0-5 COD time, IAT, back squat 1RM, and CMJ height.	Complex training.	CT significantly improved CMJ (WG d = 4.04), 1RM back squat (d = 1.21), IAT (WG d = 2.06), and 5-0-5 COD (WG d = 0.86). TRT also improved CMJ (WG d = 1.95) and 1RM back squat (WG d = 1.06), but not IAT or 5-0-5 COD. CT produced greater improvements than TRT in CMJ (partial η^2^ = 0.591) and IAT (partial η^2^ = 0.125), whereas no significant group × time interaction was observed for 1RM back squat (*p* = 0.809).
Xu et al. (2026) [28]	RCT.	*n* = 45; men; national-level track & field athletes; 19.16 ± 1.38 y.	Duration: 24 weeks; Outcome measures: Jump and drop height, countermovement impulse, push-off impulse, relative maximal force and power, countermovement and push-off time, and push-off power.	Complex training; Plyometric training.	CT significantly improved jump height (+30.9%, d = 2.09), drop height (+30.4%, d = 2.09), countermovement impulse (+24.3%), and relative maximum push-off power (+28.7%, d = 1.35). Contrast training produced improvements in push-off power (+17.1%) and reduced push-off time (−6.1%) under non-arm-swing conditions.
Kaya & Pınar (2022) [29]	Experimental study with two-group.	*n* = 16; men; athletes; ≥3 years of resistance training experience; 20.93 ± 5.54y.	Duration: 6 weeks; Outcome measures: CMJ height and RSI.	Triphasic Training; Cluster.	Within-group pre–post comparisons showed significant improvements in CMJ and RSI in both the cluster 15 s and cluster 30 s groups after 6 weeks. Cluster 30 s produced greater improvements than cluster 15 s in CMJ (+11% vs. +8%) and RSI (+11% vs. +8%), with between-group effect sizes of η^2^ = 0.35 and η^2^ = 0.29, respectively.

Note: 1RM = One-repetition maximum; AELT = Accentuated eccentric loading training; BG = between-group; CATCH = weightlifting catching derivatives; CG = Control group; CMJ = Countermovement jump; COD = Changes in direction; CT = Complex training; d = Cohen’s d; DJ-RSI = Drop jump reactive strength index; ES = Effect size; EUR = Eccentric utilization ratio; FWT = Flywheel training; G1 = Group 1; G2 = Group 2; IAT = Illinois agility test; IMTP = Isometric mid-thigh pull; IRR = Inter-repetition rest; MaxPP = Maximal peak power; MCT = Modified complex training; OL = pulling derivatives with phase-specific loading; OPL = Optimal power loading; PBRT = Percentage based resistance training; PP = peak power; PULL = weightlifting pulling derivatives; RCT = Randomized controlled trial; RFD = Rate of force development; RM = repeated-measures; RPE = Rating of perceived exertion; RSI = Reactive strength index; SJ = Squat jump; SLJ = Standing long jump; TRT = Traditional resistance training; VBT = Velocity-based training; VRT = Variable resistance training; WG = within-group; y = Years.

**Table 2:** Power Training Methods Across the Load–velocity Continuum.

Method	Typical Load Sets × Repetitions Rest Interval	Primary Objective	Exercise and Barbell Velocity (m·s^−1^) at Different %1RM	Authors
Heavy Resistance Training	80–100% 1RM 3–6 × 1–5 3–5 min ‡	Maximal strength development	BP MV: ≈0.48 (80%), ≈0.33 (90%), ≈0.19 (100%). BS MV: ≈0.46 (90%), ≈0.31 (100%). Deadlift MV: 0.51 (85%), 0.45 (90%), 0.39 (95%), 0.33 (100%).	Bird et al. (2005) [57] Benavides-Ubric et al. (2020) [58] Cormie et al. (2011) [9] González-Badillo and Sánchez-Medina (2010) [59] Sánchez-Medina et al. (2017) [60]
Ballistic Training	20–45% 1RM (or body mass) 3–6 × 1–5 3–5 min ‡	Eliminate deceleration and maximize acceleration	BPT MPV: 1.64 (20%), 1.38 (30%), 1.13 (40%), 0.93 (50%), 0.72 (60%) 0.57, (70%). JS MPV: 1.14 (24%), 0.94 (46%), 0.76 (70%), 0.61 (94%).	Cormie et al. (2011) [9] García-Ramos et al. (2018) [61] Loturco et al. (2023) [62] Lorenz and Morrison (2015) [38]
Olympic Weightlifting	70–80% 1RM 3–6 × 2–5 2–4 min ‡	Develop high power through triple extension	Jump Shrug MPV: 1.49–1.04 (20–100%), Hang High Pull MPV: 1.79–1.05 (20–100%), Hang Power Clean MPV: 1.46–1.15 (20–80%), Hang Clean Pull MPV: 1.33–1.00 (20–100%), Countermovement Shrug MPV: 1.37–1.05 (20–100%).	Cormie et al. (2011) [9] Kawamori et al. (2005) [63] Lorenz and Morrison (2015) [38] Suchomel et al. (2025) [64]
Plyometric Training	0–30% 1RM (or body mass) 3–6 × 5–15 contacts 2–3 min ‡	Improve stretch-shortening cycle efficiency and reactive strength	Exercise-dependent ‡	Moir et al. (2018) [65] Sammoud et al. (2022) [43]
Eccentric Overload Training	100–120% eccentric/70–90% concentric 3–5 × 3–6 3–5 min ‡	Increase eccentric force production, braking capacity and improve force transfer to high-velocity concentric.		Suchomel et al. (2019) [66] Wagle et al. (2017) [48]
Isometric Training	Maximal voluntary effort 3–5 contractions × 3–10 s each 2–3 min ‡	Increase maximal force and neural drive	Not applicable †	Cormie et al. (2011) [12] Ghayomzadeh et al. (2025) [67]
Variable Resistance Training	50–85% 1RM + elastic bands or chains (contributing 10–30% of the total resistance) 3–5 × 3–6 2–3 min ‡	Match resistance to the strength curve and maintain acceleration	Exercise-dependent ‡	Bird et al. (2005) [57] Suchomel et al. (2016) [68] Peng et al. (2025) [19]
Velocity-Based Training	Velocity zone (≈0.90–1.60 m·s^−1^ for power exercises) 3–5 × 1–5 2–3 min ‡	Individualized training intensity	Deadlift MV: 1.02 (40%), 0.91 (50%), 0.80 (60%), 0.68 (70%), 0.51 (80%), 0.45 (90%). BP MV: ≈1.33 (30%), ≈1.15 (40%), ≈0.97 (50%), ≈0.80 (60%), ≈0.64 (70%), ≈0.48 (80%), ≈0.33 (90%), ≈0.19 (100%). BS MV: 0.96 (40%), 0.78 (60%), 0.70 (70%), 0.61 (80%), 0.49 (90%)	Benavides-Ubric et al. (2020) [58] González-Badillo and Sánchez-Medina (2010) [59] Li et al. (2025) [69] Loturco et al. (2017) [70]
Optimal Power Load	Individual load producing maximal power (30–80%1RM depending on the exercise) 3–6 × 3–6 2–3 min ‡	Maximize mechanical power output	Half squat MV: 0.93 (32.4%), BP MV: 1.40 (27.7%), JS MV: 1.02 (28.2%), BPT MV: 1.62 (23.3%).	Bird et al. (2005) [57] Cormier et al. (2024) [8] Loturco et al. (2017) [70].
Cluster Training	70–90% 1RM 3–6 × (4–6 repetitions with 10–30 s intra-set rest every 1–2 repetitions) 2–4 min between sets ‡	Maintain movement velocity and power while reducing fatigue	Exercise-dependent ‡	Hardee et al. (2012) [18] Tufano et al. (2017) [52]
Complex-Contrast Training	80–90% 1RM + high velocity exercises 3–6 × 3–6 2–3 min ‡	Enhance power performance through complementary strength and high-velocity	Exercise-dependent ‡	Tan et al. (2026) [10] Thapa et al. (2024) [53]
Triphasic Training	70–100% 1RM Eccentric: 0–6 s lowering Isometric: 0–4 s pause Concentric: Maximal intent 3–5 × 2–5 2–4 min ‡	Sequentially develop eccentric strength, isometric force, concentric power, and neuromuscular efficiency.	Exercise-dependent ‡	Dietz and Peterson (2012) [55] Kaya & Pınar (2022) [29]

**Note:** The loading, repetition, rest, and velocity characteristics should be interpreted as general examples of possible implementation rather than universal prescription thresholds. Velocity values represent approximate values reported in the literature and should not be interpreted as directly comparable or as universal thresholds across different exercises and training methods. † Directly reported or adapted from the cited primary study/studies. ‡ Evidence-informed representative range synthesized from multiple sources and intended as practical guidance; values should not be interpreted as originating from a single study.

**Table 3:** Representative exercises for the principal resistance training methods used to develop muscular power.

Method	Exercises
Heavy Resistance Training	Back Squat; Conventional Deadlift; Barbell Bench Press.
Ballistic Training	Jump Squat; Bench Throw (Smith Machine); Medicine Ball Chest Throw.
Olympic Weightlifting	Power Clean; Hang Power Clean; Snatch Pull.
Plyometric Training	Countermovement Jump; Drop Jump; Bounding.
Optimal Power Load (OPL)	Jump Squat at OPL; Bench Press at OPL; Trap Bar Jump at OPL.
Velocity-Based Training (VBT)	Back Squat with Velocity Monitoring; Bench Press with Velocity Monitoring; Trap Bar Deadlift with Velocity Monitoring.
Cluster Sets Training	Back Squat (Cluster Configuration); Bench Press (Cluster Configuration); Unilateral Lunge (Cluster Configuration).
Variable Resistance Training	Back Squat with Elastic Bands; Bench Press with Chains; Deadlift with Elastic Bands.
Isometric Training	Isometric Mid-Thigh Pull; Isometric Squat; Isometric Bench Press.
Eccentric Overload Training	Dumbbell-Release Squat; Dumbbell-Release Lunge.
Complex-Contrast Training	Back Squat + Countermovement Jump; Bench Press + Medicine Ball Chest Throw; Trap Bar Deadlift + Jump Squat.
Triphasic Training	Bench Press eccentric emphasis: 5 s eccentric → 0 s isometric → maximal intent concentric; Bench Press isometric emphasis: 0 s eccentric → 3 s isometric → maximal intent concentric; Bench Press concentric emphasis: 0 s eccentric → 0 s isometric → maximal intent concentric.

**Notes:** Exercises are representative examples only and should be selected according to the athlete’s characteristics, sport-specific demands, and training objectives. For Triphasic Training examples, “0 s” indicates that no deliberate temporal emphasis is prescribed for that phase, rather than the complete absence of the respective movement phase.

## Data Availability

No new data were created or analyzed in this study. Data sharing is not applicable to this article.

## Supplementary Materials

The following supporting information can be downloaded at: https://www.mdpi.com/article/10.3390/sports14100464/s1, Figure S1: Literature screening and selection flow diagram; Table S1: Complete search strategies for PubMed, SPORTDiscus, and Scopus.

## References

[1] Stone M.H., Moir G., Glaister M., Sanders R. (2002). How much strength is necessary?. Phys. Ther. Sport.

[2] Taber C.B., Bellon C., Abbott H., Bingham G.E. (2016). Roles of maximal strength and rate of force development in maximizing muscular power. Strength Cond. J..

[3] Hartmann H., Bob A., Wirth K., Schmidtbleicher D. (2009). Effects of different periodization models on rate of force development and power ability of the upper extremity. J. Strength Cond. Res..

[4] Loturco I., Pereira L.A., Pareja-Blanco F. (2026). Training at different velocity zones: Does it really matter?. Int. J. Sports Physiol. Perform..

[5] Stone M.H., Stone M., Sands W.A. (2007). Principles and Practice of Resistance Training.

[6] James L.P., Haff G.G., Kelly V.G., Connick M., Hoffman B.W., Beckman E.M. (2018). The impact of strength level on adaptations to combined weightlifting, plyometric and ballistic training. Scand. J. Med. Sci. Sports.

[7] Winter E.M., Abt G., Brookes F.B., Challis J.H., Fowler N.E., Knudson D.V., Knuttgen H.G., Kraemer W.J., Lane A.M., van Mechelen W. (2016). Misuse of “power” and other mechanical terms in sport and exercise science research. J. Strength Cond. Res..

[8] Cormier P., Freitas T.T., Seaman K. (2024). A systematic review of resistance training methodologies for the development of lower body concentric mean power, peak power, and mean propulsive power in team-sport athletes. Sports Biomech..

[9] Cormie P., McGuigan M.R., Newton R.U. (2011). Developing maximal neuromuscular power: Part 2—Training considerations for improving maximal power production. Sports Med..

[10] Tan H., Yan Z., Shao C., Lin Y. (2026). Effects of different training on lower limb explosive power in youth soccer players: A systematic review and network meta-analysis. Front. Physiol..

[11] Pillitteri G., Clemente F.M., Moran J., Oliveira R., Silva R.M., Sarmento H., Puleo G., Bongiovanni T., Petrucci M., Rodríguez Fernández A. (2026). Are there optimal strength training methods for soccer players? A narrative review of methodological and practical implications. Int. J. Sports Sci. Coach..

[12] Cormie P., McGuigan M.R., Newton R.U. (2011). Developing maximal neuromuscular power: Part 1—Biological basis of maximal power production. Sports Med..

[13] Moss B.M., Refsnes P.E., Abildgaard A., Nicolaysen K., Jensen J. (1997). Effects of maximal effort strength training with different loads on dynamic strength, cross-sectional area, load-power and load-velocity relationships. Eur. J. Appl. Physiol. Occup. Physiol..

[14] Cormie P., McGuigan M.R., Newton R.U. (2010). Adaptations in athletic performance after ballistic power versus strength training. Med. Sci. Sports Exerc..

[15] Lecce E., Romagnoli R., Maffiuletti N.A., Frinolli G., Felici F., Piacentini M.F., Bazzucchi I. (2025). In the reign of velocity: Ballistic training enhances rapid force production in chronically strength-trained athletes. Int. J. Sports Physiol. Perform..

[16] Suchomel T.J., McKeever S.M., Nolen J.D., Comfort P. (2022). Muscle architectural and force-velocity curve adaptations following 10 weeks of training with weightlifting catching and pulling derivatives. J. Sports Sci. Med..

[17] Berton R., Silva D.D.D., Santos M.L.D., Silva C.M.P.E., Tricoli V. (2022). Weightlifting derivatives vs. plyometric exercises: Effects on unloaded and loaded vertical jumps and sprint performance. PLoS ONE.

[18] Hardee J.P., Lawrence M.M., Utter A.C., Triplett N.T., Zwetsloot K.A., McBride J.M. (2012). Effect of inter-repetition rest on ratings of perceived exertion during multiple sets of the power clean. Eur. J. Appl. Physiol..

[19] Peng W., Zhang Z., Lu D., Li Z., Li G., Sun J., Li D. (2025). The impact of blood flow restriction combined with variable resistance training at different loads on lower limb strength and explosiveness in collegiate basketball players. Front. Physiol..

[20] Tsoukos A., Bogdanis G.C., Terzis G., Veligekas P. (2016). Acute improvement of vertical jump performance after isometric squats depends on knee angle and vertical jumping ability. J. Strength Cond. Res..

[21] Lum D., Comfort P., Barbosa T.M., Balasekaran G. (2022). Comparing the effects of plyometric and isometric strength training on dynamic and isometric force-time characteristics. Biol. Sport.

[22] Merrigan J.J., Tufano J.J., Falzone M., Jones M.T. (2021). Effectiveness of accentuated eccentric loading: Contingent on concentric load. Int. J. Sports Physiol. Perform..

[23] Hu Z., Liu Y., Huang K., Huang H., Zhang Y., Yuan X. (2024). Effects of inertial flywheel training vs. accentuated eccentric loading training on strength, power, and speed in well-trained male college sprinters. Life.

[24] Freitas T.T., Calleja-González J., Carlos-Vivas J., Marín-Cascales E., Alcaraz P.E. (2019). Short-term optimal load training vs. a modified complex training in semi-professional basketball players. J. Sports Sci..

[25] Rauch J.T., Loturco I., Cheesman N., Thiel J., Alvarez M., Miller N., Carpenter N., Barakat C., Velasquez G., Stanjones A. (2018). Similar strength and power adaptations between two different velocity-based training regimens in collegiate female volleyball players. Sports.

[26] Chen J., Deng B., He T., He J., Li D., Lu M., Sun J. (2025). Individualized responses to velocity-based versus percentage-based resistance training in combat sports athletes: The influence of baseline characteristics. PeerJ.

[27] Wang B., Xie E., Liang P., Liu T., Zhu J., Qin G., Su X. (2024). Transforming performance: The impact of an 8-week complex training program on strength, power, and change of direction in female basketball athletes. Medicine.

[28] Xu Z., Lin Y., Zhang F., Li Q., Yu L., Lin M., Ning F., Wang X. (2026). Effects of complex and contrast training on lower limb explosive power and eccentric strength in elite male track and field athletes: A 24-week randomized trial. J. Strength Cond. Res..

[29] Kaya S., Pınar S. (2022). Effects of triphasic training model combined with two different cluster sets on vertical jump and reactive strength index. Phys. Educ. Stud..

[30] Haff G.G., Nimphius S. (2012). Training principles for power. Strength Cond. J..

[31] Knuttgen H.G., Komi P.V., Komi P.V. (2003). Basic Considerations for Exercise. Strength and Power in Sport.

[32] Monteiro L., Massuça L.M., Ramos S., Garcia-Garcia J. (2024). Neuromuscular performance of world-class Judo athletes on bench press, prone row and repeated jump tests. Appl. Sci..

[33] Lindberg K., Solberg P.A., Bjørnsen T., Helland C., Rønnestad B.R., Frank M.T., Kristiansen E.L., Berntsen S., Haug V.B., Paulsen G. (2021). Force-velocity profiling in athletes: Reliability and agreement across methods. PLoS ONE.

[34] Banyard H.G., Nosaka K., Vernon A.D., Haff G.G. (2018). The reliability of individualized load–velocity profiles. Int. J. Sports Physiol. Perform..

[35] Torrejón A., Balsalobre-Fernández C., Haff G.G., García-Ramos A. (2019). The load-velocity profile differs more between men and women than between individuals with different strength levels. Sports Biomech..

[36] Newton R.U., Kraemer W.J., Häkkinen K., Humphries B.J., Murphy A.J. (1996). Kinematics, kinetics, and muscle activation during explosive upper body movements. J. Appl. Biomech..

[37] Dideriksen J.L., Del Vecchio A., Farina D. (2020). Neural and muscular determinants of maximal rate of force development. J. Neurophysiol..

[38] Lorenz D., Morrison S. (2015). Current concepts in periodization of strength and conditioning for the sports physical therapist. Int. J. Sports Phys. Ther..

[39] Loturco I., Dello Iacono A., Nakamura F.Y., Freitas T.T., Boullosa D., Valenzuela P.L., Pereira L.A., McGuigan M.R. (2022). The optimum power load: A simple and powerful tool for testing and training. Int. J. Sports Physiol. Perform..

[40] Marovic I., Janicijevic D., Knežević O.M., Garcia-Ramos A., Prebeg G., Mirkov D.M. (2025). Potential use of the medicine ball throw test to reveal the upper-body maximal capacities to produce force, velocity, and power. Proc. Inst. Mech. Eng. Part P J. Sports Eng. Technol..

[41] Cormie P., McBride J.M., McCaulley G.O. (2009). Power-time, force-time, and velocity-time curve analysis of the countermovement jump: Impact of training. J. Strength Cond. Res..

[42] Suchomel T.J., Comfort P., Stone M.H. (2015). Weightlifting pulling derivatives: Rationale for implementation and application. Sports Med..

[43] Sammoud S., Bouguezzi R., Ramirez-Campillo R., Negra Y., Prieske O., Moran J., Granacher U., Chaabene H. (2022). Effects of plyometric jump training versus power training using free weights on measures of physical fitness in youth male soccer players. J. Sports Sci..

[44] Komi P.V. (2008). Strength and Power in Sport.

[45] Oranchuk D.J., Storey A.G., Nelson A.R., Cronin J.B. (2019). Isometric training and long-term adaptations: Effects of muscle length, intensity, and intent: A systematic review. Scand. J. Med. Sci. Sports.

[46] de Hoyo M., Pozzo M., Sañudo B., Carrasco L., Gonzalo-Skok O., Domínguez-Cobo S., Morán-Camacho E., Otero-Esquina C., Suárez-Arrones L., Sánchez H. (2015). Effects of a 10-week in-season eccentric-overload training program on muscle-injury prevention and performance in junior elite soccer players. Int. J. Sports Physiol. Perform..

[47] Friedmann-Bette B., Bauer T., Kinscherf R., Vorwald S., Klute K., Bischoff D., Müller H., Berchtold A., Hoffmann U., Bärtsch P. (2010). Effects of strength training with eccentric overload on muscle adaptation in male athletes. Eur. J. Appl. Physiol..

[48] Wagle J.P., Taber C.B., Cunanan A.J., Bingham G.E., Carroll K.M., DeWeese B.H., Sato K., Stone M.H. (2017). Accentuated eccentric loading for training and performance: A review. Sports Med..

[49] Abián-Vicén J., Martínez F., Jiménez F., Abián P. (2022). Effects of eccentric single-leg decline squat training performed with different execution times on maximal strength and muscle contraction properties of the knee extensor muscles. J. Strength Cond. Res..

[50] Ramírez J.M., Núñez V.M., Lancho C., Poblador M.S., Lancho J.L. (2015). Velocity-based training of lower limb to improve absolute and relative power outputs in concentric phase of half-squat in soccer players. J. Strength Cond. Res..

[51] García-Ramos A., Padial P., Haff G.G., Argüelles-Cienfuegos J., García-Ramos M., Conde-Pipó J., Feriche B. (2015). Effect of different interrepetition rest periods on barbell velocity loss during the ballistic bench press exercise. J. Strength Cond. Res..

[52] Tufano J.J., Brown L.E., Haff G.G. (2017). Theoretical and practical aspects of different cluster set structures: A systematic review. J. Strength Cond. Res..

[53] Thapa R.K., Weldon A., Freitas T.T., Boullosa D., Afonso J., Granacher U., Ramirez-Campillo R. (2024). What do we know about complex-contrast training? A systematic scoping review. Sports Med. Open.

[54] Matthews M., Comfort P. (2008). Applying complex training principles to Boxing: A practical approach. Strength Cond. J..

[55] Dietz C., Peterson B. (2012). Triphasic Training: A Systematic Approach to Elite Speed and Explosive Strength Performance.

[56] Rebelo A., Pereira J.R., Valente-dos-Santos J. (2023). Effects of a preseason triphasic resistance training program on athletic performance in elite volleyball players—An observational study. Ger. J. Exerc. Sport Res..

[57] Bird S.P., Tarpenning K.M., Marino F.E. (2005). Designing resistance training programmes to enhance muscular fitness: A review of the acute programme variables. Sports Med..

[58] Benavides-Ubric A., Díez-Fernández D.M., Rodríguez-Pérez M.A., Ortega-Becerra M., Pareja-Blanco F. (2020). Analysis of the load-velocity relationship in deadlift exercise. J. Sports Sci. Med..

[59] González-Badillo J.J., Sánchez-Medina L. (2010). Movement velocity as a measure of loading intensity in resistance training. Int. J. Sports Med..

[60] Sánchez-Medina L., Pallarés J.G., Pérez C.E., Morán-Navarro R., González-Badillo J.J. (2017). Estimation of relative load from bar velocity in the full back squat exercise. Sports Med. Int. Open.

[61] García-Ramos A., Haff G.G., Padial P., Feriche B. (2018). Reliability of power and velocity variables collected during the traditional and ballistic bench press exercise. Sports Biomech..

[62] Loturco I., McGuigan M., Pereira L.A., Pareja-Blanco F. (2023). The load-velocity relationship in the jump squat exercise. Biol. Sport.

[63] Kawamori N., Crum A.J., Blumert P.A., Kulik J., Childers J., Wood J.A., Stone M.H., Haff G.G. (2005). Influence of different relative intensities on power output during the hang power clean: Identification of the optimal load. J. Strength Cond. Res..

[64] Suchomel T.J., Kissick C.R., Techmanski B.S., Mann J.B., Comfort P. (2025). Velocity-based training with weightlifting derivatives: Barbell and system velocity comparisons. J. Strength Cond. Res..

[65] Moir G.L., Snyder B.W., Connaboy C., Lamont H.S., Davis S.E. (2018). Using drop jumps and jump squats to assess eccentric and concentric force-velocity characteristics. Sports.

[66] Suchomel T.J., Wagle J.P., Douglas J., Taber C.B., Harden M., Haff G.G., Stone M.H. (2019). Implementing eccentric resistance training—Part 2: Practical recommendations. J. Funct. Morphol. Kinesiol..

[67] Ghayomzadeh M., Li J., Eidy F., Keshavarz M., Sabag A., Fornusek C., Hackett D.A. (2025). Effects of isometric vs. dynamic resistance training on isometric and isokinetic muscular strength: A systematic review with meta-analysis. J. Sci. Med. Sport.

[68] Suchomel T.J., Lamont H.S., Moir G.L. (2016). Understanding vertical jump potentiation: A deterministic model. Sports Med..

[69] Li Z., Xiao L., Zhang X., Lu C., Bai J. (2025). Optimization of applied loads for assessing load-velocity relationship during back squat. PLoS ONE.

[70] Loturco I., Pereira L.A., Abad C.C.C., Tabares F., Moraes J.E., Kobal R., Kitamura K., Nakamura F.Y. (2017). Bar velocities capable of optimising the muscle power in strength-power exercises. J. Sports Sci..

[71] Swinton P.A., Schoenfeld B.J., Murphy A. (2024). Dose–response modelling of resistance exercise across outcome domains in strength and conditioning: A meta-analysis. Sports Med..

[72] Androulakis-Korakakis P., Fisher J.P., Steele J. (2020). The minimum effective training dose required to increase 1RM strength in resistance-trained men: A systematic review and meta-analysis. Sports Med..

[73] Wellman A.D., Coad S.C., Flynn P.J., Siam T.K., McLellan C.P. (2019). Comparison of preseason and in-season practice and game loads in National Collegiate Athletic Association Division I football players. J. Strength Cond. Res..

[74] Fisher J.P., Csapo R. (2021). Periodization and programming in sports. Sports.

[75] Jeong T.S., Reilly T., Morton J., Bae S.W., Drust B. (2011). Quantification of the physiological loading of one week of “pre-season” and one week of “in-season” training in professional soccer players. J. Sports Sci..

[76] Issurin V.B. (2010). New horizons for the methodology and physiology of training periodization. Sports Med..

[77] Kataoka R., Vasenina E., Loenneke J., Buckner S.L. (2021). Periodization: Variation in the definition and discrepancies in study design. Sports Med..

[78] Mølmen K.S., Øfsteng S.J., Rønnestad B.R. (2019). Block periodization of endurance training—A systematic review and meta-analysis. Open Access J. Sports Med..

