# Supplementary material for: Power Training Methods Across the Load–Velocity Continuum: Mechanisms, Applications, and Periodization
Source: Sports (Basel). 2026 Oct 20;14(10):464. doi: 10.3390/sports14100464 (PMC13709544; doi:10.3390/sports14100464)
Supplement: Supplementary file 1 [file sports-14-00464-s001.zip › sports-4583973-supplementary.pdf]

**Supplementary Table S1.** Complete Search Strategies for PubMed, SPORTDiscus, and Scopus		
**Database**	**Search strategy**	**Limits**
**PubMed**	("muscular power"[Title/Abstract] OR "power training"[Title/Abstract]) AND ("strength training"[Title/Abstract] OR "maximal strength training"[Title/Abstract] OR "heavy resistance training"[Title/Abstract] OR "isometric training"[Title/Abstract] OR "Olympic weightlifting"[Title/Abstract] OR "weightlifting derivatives"[Title/Abstract] OR "ballistic training"[Title/Abstract] OR "plyometric training"[Title/Abstract] OR "velocity-based training"[Title/Abstract] OR "optimal power load"[Title/Abstract] OR "variable resistance training"[Title/Abstract] OR "cluster sets"[Title/Abstract] OR "eccentric training"[Title/Abstract] OR "complex training"[Title/Abstract] OR "triphasic training"[Title/Abstract]) AND English[Language]	English; no publication-date restriction
**SPORTDiscus**	XB ("muscular power" OR "power training") AND XB ("strength training" OR "maximal strength training" OR "heavy resistance training" OR "isometric training" OR "Olympic weightlifting" OR "weightlifting derivatives" OR "ballistic training" OR "plyometric training" OR "velocity-based training" OR "optimal power load" OR "variable resistance training" OR "cluster sets" OR "eccentric training" OR "complex training" OR "triphasic training")	English; no publication-date restriction
**Scopus**	(TITLE-ABS-KEY("muscular power" OR "power training") AND TITLE-ABS-KEY("strength training" OR "maximal strength training" OR "heavy resistance training" OR "isometric training" OR "Olympic weightlifting" OR "weightlifting derivatives" OR "ballistic training" OR "plyometric training" OR "velocity-based training" OR "optimal power load" OR "variable resistance training" OR "cluster sets" OR "eccentric training" OR "complex training" OR "triphasic training") AND (LIMIT-TO (LANGUAGE , "English"))	English; no publication-date restriction

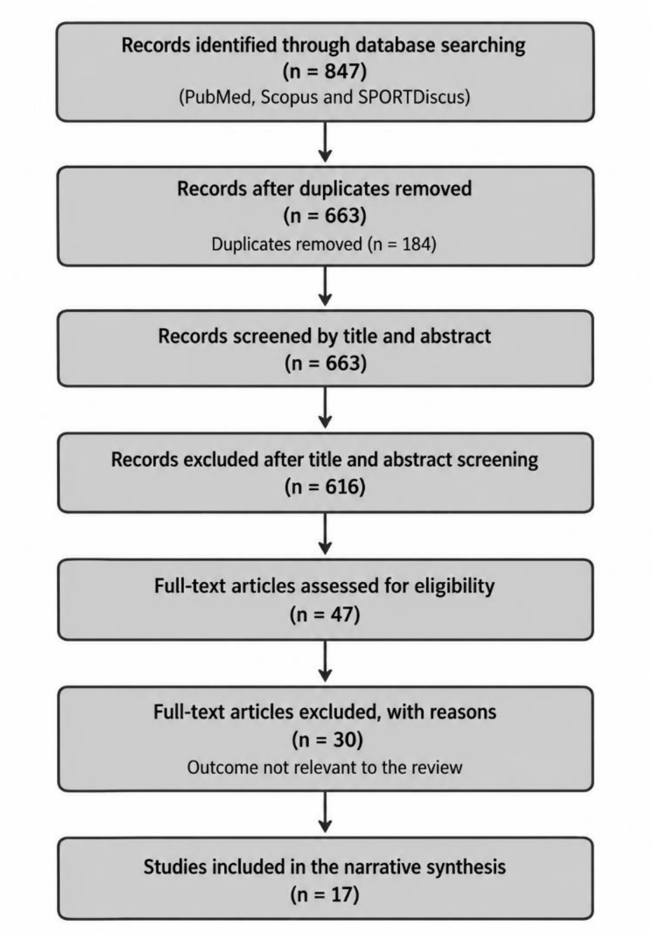

Supplementary Figure S1. Literature screening and selection flow diagram. A total of 847 records were identified through database searching, including 239 records from PubMed, 208 from SPORTDiscus, and 400 from Scopus. After removal of 184 duplicates, 663 records were screened based on title and abstract, of which 616 were excluded. The remaining 47 articles were assessed in full text for eligibility. Following full-text assessment, 30 articles were excluded because their outcomes did not address the objective of the review. A total of 17 studies were included in the narrative synthesis.
